# Dual oxidase 2 and pancreatic adenocarcinoma: IFN-γ-mediated dual oxidase 2 overexpression results in H_2_O_2_-induced, ERK-associated up-regulation of HIF-1α and VEGF-A

**DOI:** 10.18632/oncotarget.12032

**Published:** 2016-09-15

**Authors:** Yongzhong Wu, Jennifer L. Meitzler, Smitha Antony, Agnes Juhasz, Jiamo Lu, Guojian Jiang, Han Liu, Melinda Hollingshead, Diana C. Haines, Donna Butcher, Michaela S. Panter, Krishnendu Roy, James H. Doroshow

**Affiliations:** ^1^ Center for Cancer Research, National Cancer Institute, Bethesda, MD, USA; ^2^ Division of Cancer Treatment and Diagnosis, National Cancer Institute, Bethesda, MD, USA; ^3^ Pathology/Histotechnology Laboratory, Leidos, Inc./Frederick National Laboratory for Cancer Research, National Cancer Institute, Frederick, MD, USA

**Keywords:** dual oxidase, NADPH oxidase, pancreatic cancer, hydrogen peroxide, angiogenesis

## Abstract

Several NADPH oxidase family members, including dual oxidase 2 [DUOX2], are expressed in human tumors, particularly gastrointestinal cancers associated with long-standing chronic inflammation. We found previously that exposure of pancreatic ductal adenocarcinoma cells to the pro-inflammatory cytokine IFN-γ increased DUOX2 expression (but not other NADPH oxidases) leading to long-lived H_2_O_2_ production. To elucidate the pathophysiology of DUOX2-mediated H_2_O_2_ formation in the pancreas further, we demonstrate here that IFN-γ-treated BxPC-3 and CFPAC-1 pancreatic cancer cells (known to increase DUOX2 expression) produce significant levels of intracellular oxidants and extracellular H_2_O_2_ which correlate with concomitant up-regulation of VEGF-A and HIF-1α transcription. These changes are not observed in the PANC-1 line that does not increase DUOX2 expression following IFN-γ treatment. DUOX2 knockdown with short interfering RNA significantly decreased IFN-γ-induced VEGF-A or HIF-1α up-regulation, as did treatment of pancreatic cancer cells with the NADPH oxidase inhibitor diphenylene iodonium, the multifunctional reduced thiol N-acetylcysteine, and the polyethylene glycol-modified form of the hydrogen peroxide detoxifying enzyme catalase. Increased DUOX2-related VEGF-A expression appears to result from reactive oxygen-mediated activation of ERK signaling that is responsible for AP-1-related transcriptional effects on the VEGF-A promoter. To clarify the relevance of these observations *in vivo*, we demonstrate that many human pre-malignant pancreatic intraepithelial neoplasms and frank pancreatic cancers express substantial levels of DUOX protein compared to histologically normal pancreatic tissues, and that expression of both DUOX2 and VEGF-A mRNAs is significantly increased in surgically-resected pancreatic cancers compared to the adjacent normal pancreas.

## INTRODUCTION

Pancreatic ductal adenocarcinomas [PDACs] have been demonstrated to produce significantly greater levels of reactive oxygen species [ROS] than non-malignant cells from the pancreas [1, 2]. ROS production in pancreatic tumors has also been associated with several hallmarks of cancer, including: enhanced cellular proliferation [1–4]; decreased apoptosis [5–8] due, in part, to inhibition of tumor-suppressive protein tyrosine phosphatases [PTPs] [9, 10]; and increased DNA damage [11]. ROS formation in the pancreas can be induced by pro-inflammatory cytokines, such as interferon-γ [IFN-γ] [11, 12], as well as by growth factors and oncogene expression [1]. Thus, it is possible that chronic inflammation could accelerate malignant transformation and tumor progression in the pancreas by increasing the cell proliferation and genomic instability that arise during repeated episodes of cytokine-induced inflammatory stress [13, 14].

ROS production in many epithelial malignancies, including PDAC, may result from the activity of one or more members of the NADPH oxidase [Nox] gene family. The expression of these enzymes has been shown to be significantly up-regulated in many human cancer cell lines and in human tumors (when expression levels in cancers are compared to adjacent non-malignant tissues) [15]. In particular, the H_2_O_2_-generating Nox isoform dual oxidase 2 [DUOX2] can be highly expressed in patients with chronic pancreatitis [11, 16], or PDAC [16], as well as in human pancreatic cancer xenografts in mice [11]. Furthermore, expression of the *DUOX2* gene and protein is increased in various human pancreatic cancer cell lines following IFN-γ and/or lipopolysaccharide [LPS] stimulation [11, 12, 17].

Similar to the other five Nox isoforms, DUOX1 and DUOX2 are glycoproteins consisting of six transmembrane helices bearing a cytosolic C-terminal FAD/NADPH binding domain. However, the DUOX proteins also encompass a distinctive extracellular N-terminal peroxidase-like domain that is anchored in the membrane by a seventh transmembrane helix and two cytosolic calcium-binding sites. Together, these structural components mediate the transfer of electrons from NADPH to molecular oxygen to produce H_2_O_2_. Among its specific interaction partners, DUOX2 requires the maturation factor DUOXA2 for the formation of a functional, H_2_O_2_-producing complex; the expression of DUOXA2, like DUOX2, is also up-regulated by IFN-γ exposure in human pancreatic cancer cells [12, 17]. To date, DUOX2 has primarily been investigated to determine its role in the production of the H_2_O_2_ required for thyroid hormone biosynthesis [18] and to elucidate how it functions as a component of mucosal host defense systems, particularly in the gastrointestinal and respiratory tracts [19, 20]. However, recent studies have demonstrated that marked DUOX2 overexpression is distributed across a range of human solid tumors [17]. Hence, understanding whether and how DUOX2-related H_2_O_2_ formation plays a role in the pathogenesis of human malignancies associated with inflammation has become an area of active investigation.

Resistance to apoptosis by cancer cells is a hallmark of tumor cell growth and progression. In pancreatic cancer cells, apoptotic resistance is modulated not only by Nox-generated ROS but also by hypoxia-inducible factor-1α [HIF-1α] [21], a redox-sensitive transcription factor that is overexpressed in pancreatic carcinoma relative to adjacent normal pancreatic tissue [22]. HIF-1α expression in PDAC is also associated with increased expression of vascular endothelial growth factor [VEGF] [23]. In turn, VEGF expression has been linked to pancreatic tumor stage and local disease progression [24].

The expression levels of Nox and VEGF have previously been associated with certain types of human malignancies [25, 26]. In particular, superoxide produced by Nox1 have been demonstrated to trigger the development of an angiogenic phenotype, which includes VEGF production, in oncogene-transformed human fibroblasts and in human prostate cancer cells [27]. p22^phox^, a critical subunit of several Nox isoforms (Nox1-4), up-regulates HIF-1α and VEGF expression through Akt and ERK signaling in human prostate cancer [28]. Hydrogen peroxide derived from the activity of Nox4 has also been reported to stimulate HIF-1α-mediated VEGF expression in human ovarian and renal cancer cells [29, 30]. However, a relationship between the expression of the DUOX isoforms and VEGF in human cancers remains uncharacterized.

In this study, we found that increased DUOX2 expression was associated with a significant increase in the expression of the pro-angiogenic proteins, HIF-1α and VEGF-A, in human pancreatic cancer cells. Signaling that originated, at least in part, from DUOX2-mediated H_2_O_2_ production was responsible for ERK-related activation of activator protein 1 [AP-1], which appeared to play a role in the up-regulation of VEGF-A. Significant increases in DUOX2 and VEGF-A mRNA expression were demonstrated in surgically-resected human pancreatic cancer specimens compared to adjacent normal pancreatic tissues. Furthermore, increased levels of DUOX protein were demonstrable by immunohistochemistry in many PDACs and all specimens of pre-malignant pancreatic intraepithelial neoplasia [PanIN] compared to the normal pancreas. Finally, the expression of both DUOX2 and VEGF-A was rapidly increased when human pancreatic cancer cells were propagated as xenografts in immunocompromised mice. These results suggest that the production of H_2_O_2_ by DUOX2 could contribute to the inflammatory stress accompanying the development and progression of human pancreatic cancers.

## RESULTS

### VEGF-A transcription is increased in IFN-γ-stimulated pancreatic cancer cell lines that demonstrate increased DUOX2 expression

**Figure 1:**
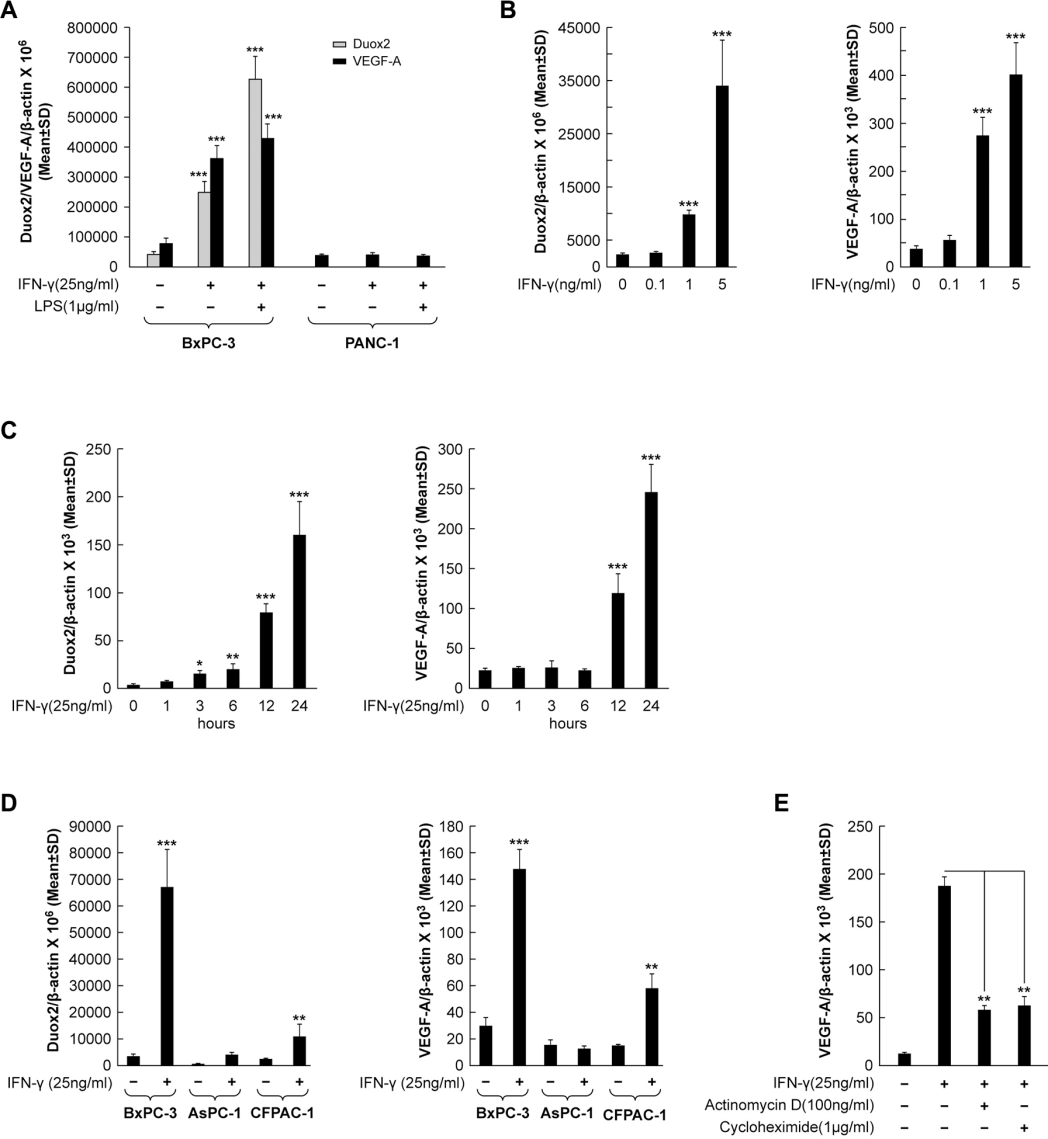
VEGF-A transcription and synthesis are up-regulated in IFN-γ-stimulated pancreatic cancer cell lines that demonstrate increased DUOX2 expression following cytokine exposure (**A**) DUOX2 and VEGF-A expression in BxPC-3 and PANC-1 cells following exposure to IFN-γ or IFN-γ and LPS for 24 h, as determined by quantitative PCR. ***P < 0.001 vs. solvent-treated cells. (**B**) Dose-response effect of IFN-γ concentration on DUOX2 (left) and VEGF-A (right) expression in BxPC-3 cells following exposure for 24 h, as determined by quantitative RT-PCR. ***P < 0.001 vs. solvent-treated cells. (**C**) Time course of IFN-γ-mediated induction of DUOX2 (left) and VEGF-A (right) expression in BxPC-3 cells, as determined by quantitative RT-PCR. *P < 0.05, **P < 0.01, ***P < 0.001 vs. cells at 0 h. (**D**) DUOX2 (left) and VEGF-A (right) expression in BxPC-3, AsPC-1, and CFPAC-1 cells following exposure to IFN-γ for 24 h, as determined by quantitative RT-PCR. **P < 0.01, ***P < 0.001 vs. solvent-treated cells. (**E**) VEGF-A expression in BxPC-3 cells pretreated with the transcription inhibitor actinomycin D or the protein synthesis inhibitor cycloheximide for 30 min, followed by IFN-γ treatment for 24 h, as determined by quantitative RT-PCR. **P < 0.01 vs. IFN-γ-stimulated, non-inhibitor-treated cells. In all panels, the data are expressed as the mean ± SD of at least three independent experiments.

We previously reported that several human pancreatic cancer cell lines up-regulate the expression of DUOX2 and DUOXA2 in response to treatment with the pro-inflammatory cytokines IFN-γ and LPS, although to varying degrees [11]. In the present study, we expanded our investigations to include VEGF-A, and found that VEGF-A and DUOX2 levels are positively associated under these circumstances. As shown in Figure 1A, IFN-γ significantly induced both DUOX2 and VEGF-A transcription in the BxPC-3 pancreatic cancer cell line (*P* < 0.001 vs. solvent-treated cells). The combination of IFN-γ and LPS resulted in further up-regulation of DUOX2, as shown previously [11]. In contrast, although the BxPC-3 and PANC-1 lines have comparable basal levels of VEGF-A, no effect on VEGF-A expression was observed in PANC-1cells treated with IFN-γ or the combination of IFN-γ and LPS; this result is consistent with our previous finding that DUOX2 expression is not inducible by IFN-γ in this cell line [12]. A dose-response evaluation (Figure 1B) demonstrated that as little as 1 ng/ml IFN-γ significantly up-regulated both DUOX2 and VEGF-A expression in BxPC-3 cells, and that 5 ng/ml IFN-γ had a stronger effect (*P* < 0.001 vs. solvent-treated cells in all cases). The time course of treatment with IFN-γ (25 ng/ml; Figure 1C) revealed that following 3 h of treatment, DUOX2 expression was significantly up-regulated in this cell type, with a progressive increase from 3 h to 24 h (*P* < 0.001 at 24 h vs. cells at 0 h). However, VEGF-A expression exhibited a delayed response to IFN-γ stimulation, with significant up-regulation occurring at 12 h; an ≈ 10-fold increase in VEGF-A levels relative to basal expression was noted at 24 h (*P* < 0.001 vs. cells at 0 h in both cases). Taken together, these results demonstrated that DUOX2 expression and VEGF-A expression are up-regulated in concert in IFN-γ-responsive pancreatic cancer cells.

To evaluate whether VEGF-A expression is increased in other human pancreatic cancer cell lines following stimulation with IFN-γ and subsequent up-regulation of DUOX2, we compared BxPC-3 cells with the AsPC-1 and CFPAC-1 lines (Figure 1D). We found that IFN-γ stimulation significantly increased DUOX2 and VEGF-A expression in CFPAC-1 cells (*P* < 0.01 in both cases), although not to the same extent observed in BxPC-3 cells (*P* < 0.001). In contrast, the AsPC-1 line demonstrated a lesser change in DUOX2 expression in response to IFN-γ. These findings are consistent with the results presented in Figure 1A, demonstrating that VEGF-A expression increases only in pancreatic cancer cell lines that up-regulate DUOX2 in response to IFN-γ stimulation.

Next, we sought to determine the underlying molecular mechanism behind cytokine-mediated VEGF-A up-regulation in human pancreatic cancer cells. As shown in Figure 1E, treatment of IFN-γ-stimulated BxPC-3 cells with the transcription inhibitor actinomycin D resulted in a significant decrease in VEGF-A expression (*P* < 0.01 vs. IFN-γ-stimulated, non-inhibitor-treated cells). This finding indicates that IFN-γ stimulates transcriptional up-regulation of VEGF-A. However, exposure to the protein synthesis inhibitor cycloheximide also produced a significant decrease in VEGF-A mRNA levels (*P* < 0.01 vs. IFN-γ-stimulated, non-inhibitor-treated cells), suggesting that VEGF-A up-regulation by cytokines is not only transcriptionally up-regulated, but that transcriptional up-regulation depends on new protein synthesis in IFN-γ-stimulated BxPC-3 cells (Figure 1E).

### HIF-1α synthesis is up-regulated in IFN-γ-stimulated pancreatic cancer cell lines that exhibit increased DUOX2 expression and function following cytokine treatment

**Figure 2:**
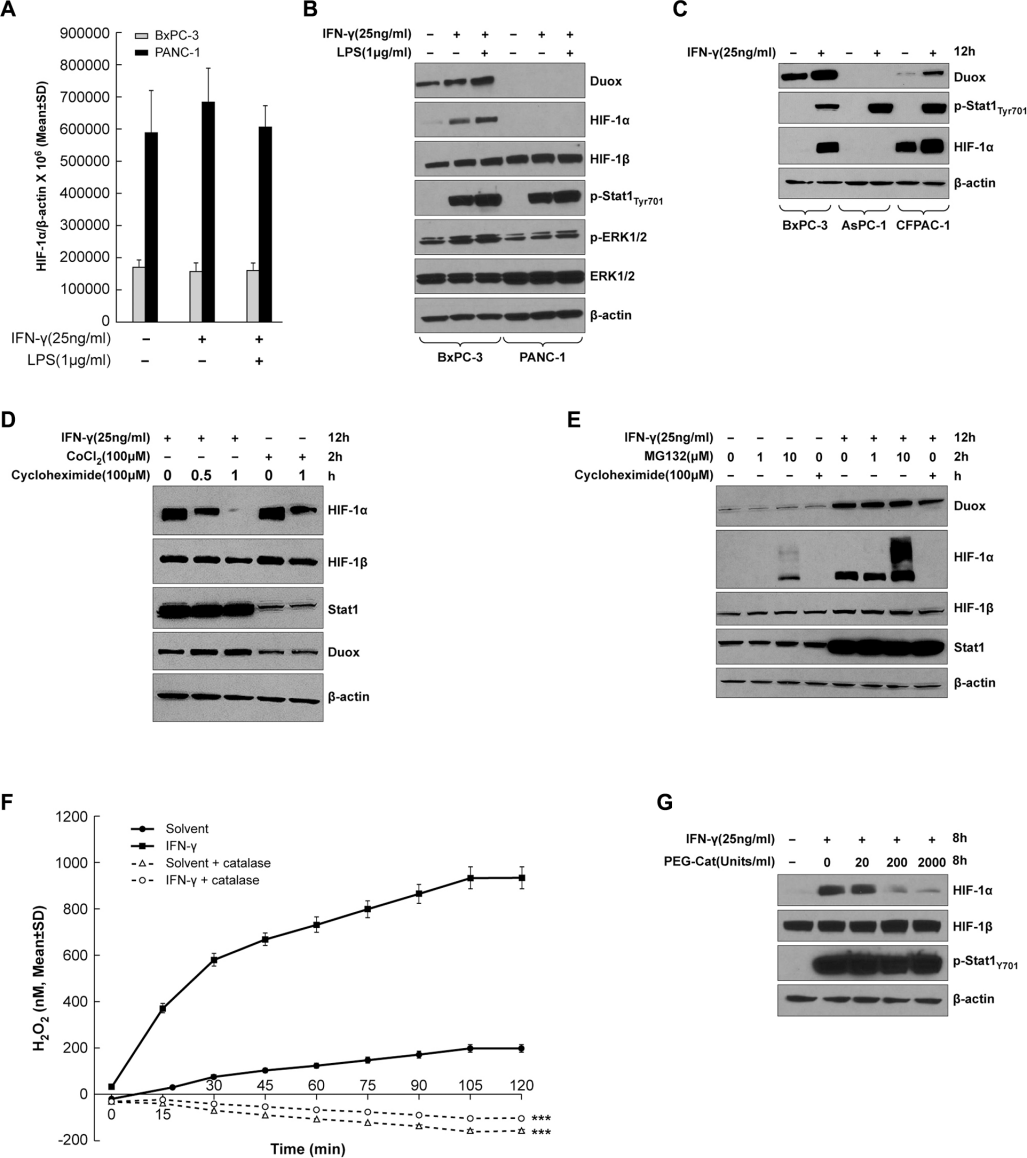
HIF-1α synthesis is up-regulated in IFN-γ-stimulated pancreatic cancer cell lines that exhibit increased DUOX2 expression following cytokine treatment (**A**) HIF-1α expression in BxPC-3 and PANC-1 cells treated with IFN-γ or the combination of IFN-γ and LPS for 24 h, as determined by quantitative RT-PCR. (**B**) Western analysis of WCEs from BxPC-3 and PANC-1 cells treated with IFN-γ or the combination of IFN-γ and LPS for 12 h. (**C**) Western analysis of WCEs from BxPC-3, AsPC-1, and CFPAC-1 cells treated with IFN-γ for 12 h. (**D**) Western analysis of WCEs from BxPC-3 cells treated with IFN-γ for 12 h or with cobalt chloride [CoCl_2_], a chemical inducer of HIF-1α, for 2 h, followed by treatment with the protein synthesis inhibitor cycloheximide for the indicated times. (**E**) Western analysis of WCEs from BxPC-3 cells treated with or without IFN-γ for 12 h, followed by treatment with the proteasome inhibitor MG132 at the indicated concentrations or with cycloheximide [Chx] for 2 h. (**F**) BxPC-3 cells were treated with IFN-γ (50 ng/ml) or solvent for 24 h, then washed with PBS, and assayed with the Amplex Red^®^ reagent for extracellular H_2_O_2_ production in the presence of ionomycin (1 μM) with or without PEG-catalase (1000 units/ml). (**G**) Western analysis of WCEs from BxPC-3 cells treated with or without IFN-γ for 8 h in the absence or presence of increasing concentrations of PEG-catalase. In B-E and G, β-actin served as a loading control. The data are expressed as the mean ± SD of at least three independent experiments (A, F) or are representative of at least three independent experiments (B–E and G). ***P < 0.001 vs. cells without PEG-catalase.

We next studied whether HIF-1α, a transcriptional regulator of VEGF-A, is also up-regulated in cytokine-stimulated pancreatic cancer cell lines. As demonstrated in Figure 2A, IFN-γ alone or combined with LPS treatment did not significantly affect the expression of HIF-1α at the mRNA level in either BxPC-3 or PANC-1 cells, indicating that DUOX2 and VEGF-A up-regulation (as demonstrated following cytokine exposure in BxPC-3 cells; Figure 1A) is not associated with changes in HIF-1α transcription. We then examined HIF-1α protein expression in the same cell lines (Figure 2B). IFN-γ stimulation or combined exposure to IFN-γ and LPS in BxPC-3 cells markedly increased HIF-1α protein levels. DUOX protein was also up-regulated, with the highest level induced by the combination of IFN-γ and LPS. Unlike BxPC-3 cells, for the PANC-1 line neither DUOX nor HIF-1α protein expression was observed under any condition, despite the presence of constitutive levels of HIF-1β, which is the other of the two subunits of HIF-1, and despite similar levels of Stat1 activation (at Tyr701) by IFN-γ in both cell lines. We also found that IFN-γ stimulation led to increases in DUOX and HIF-1α protein in CFPAC-1 as well as BxPC-3 cells, but not in the AsPC-1 line despite increased Stat1 tyrosine phosphorylation in all cell lines following IFN-γ treatment (Figure 2C). Therefore, it appears that pancreatic cancer cells that increase DUOX protein expression in response to IFN-γ up-regulate HIF-1α protein.

HIF-1α can be regulated at the level of protein stability or synthesis. In particular, under hypoxic conditions, where O_2_ concentration becomes limiting, prolyl hydroxylation and ubiquitin-mediated proteasomal degradation of HIF-1α are suppressed, resulting in HIF-1α accumulation and stabilization by dimerization with HIF-1β [31]. In contrast, cytokines, growth factors, hormones, oncogenes, and ROS can also activate certain signaling pathways to enhance HIF-1α synthesis [32–34]. To determine which of these mechanisms is involved in IFN-γ-mediated HIF-1α protein accumulation in the BxPC-3 line, we treated cells with either IFN-γ or cobalt chloride, a chemical inducer of HIF-1α, to trigger HIF-1α protein accumulation, followed by treatment with cycloheximide to stop ongoing protein synthesis (Figure 2D). HIF-1α accumulation was initially comparable in IFN-γ-treated and cobalt chloride-treated cells; however, after 1 h of cycloheximide exposure, this accumulation almost disappeared in the IFN-γ-treated tumor cells, suggesting that the stimulatory effect of IFN-γ on HIF-1α protein expression in BxPC-3 cells depends on protein synthesis, and not simply on increased protein stability. In contrast, cobalt chloride-treated cells exhibited HIF-1α protein accumulation following cycloheximide treatment, a result consistent with the expected stabilization of HIF-1α. Additionally, IFN-γ-stimulated BxPC-3 cells demonstrated similar levels of HIF-1α protein accumulation before and after treatment with a low concentration of the proteasome inhibitor MG132 (Figure 2E); only a high concentration of MG132 produced an increase in HIF-1α protein. In the same experiment, HIF-1α protein accumulation was abolished by cycloheximide. These results suggest that IFN-γ-stimulated HIF-1α accumulation in BxPC-3 cells may primarily be due to enhanced HIF-1α protein synthesis, and not to decreased degradation coupled with increased HIF-1α stability.

Finally, we examined the relationship between the production of H_2_O_2_ by IFN-γ-treated BxPC-3 cells and the expression of HIF-1α. Following treatment with solvent or 50 ng/ml of IFN-γ for 24 h, BxPC-3 cells were washed, re-suspended in glucose-containing buffer, the Amplex Red^®^ reagent, ionomycin, and either solvent or polyethylene glycol-modified catalase [PEG-catalase]. As shown in Figure 2F, in the presence of a supply of glucose in the reaction buffer—which allows continuous generation of intracellular NADPH—BxPC-3 cells treated with IFN-γ (that express enhanced levels of DUOX2) produce large amounts of extracellular H_2_O_2._ The presence of the calcium ionophore ionomycin stimulates maximal dual oxidase function [35]. The addition of PEG-catalase eliminated measurable extracellular H_2_O_2_ from both solvent-treated and IFN-γ-exposed BxPC-3 cells, *P* < 0.001 for either condition. When BxPC-3 tumor cells were treated for 8 h with IFN-γ alone or in combination with PEG-catalase (to increase intracellular peroxide detoxifying capacity) [36, 37], the induction of HIF-1α by IFN-γ was decreased in a PEG-catalase concentration-dependent fashion (Figure 2G).

### IFN-γ induces DUOX2 expression, ERK signaling, and HIF-1α synthesis in BxPC-3 cells in a fashion that resembles the effect of exogenous H_2_O_2_

**Figure 3:**
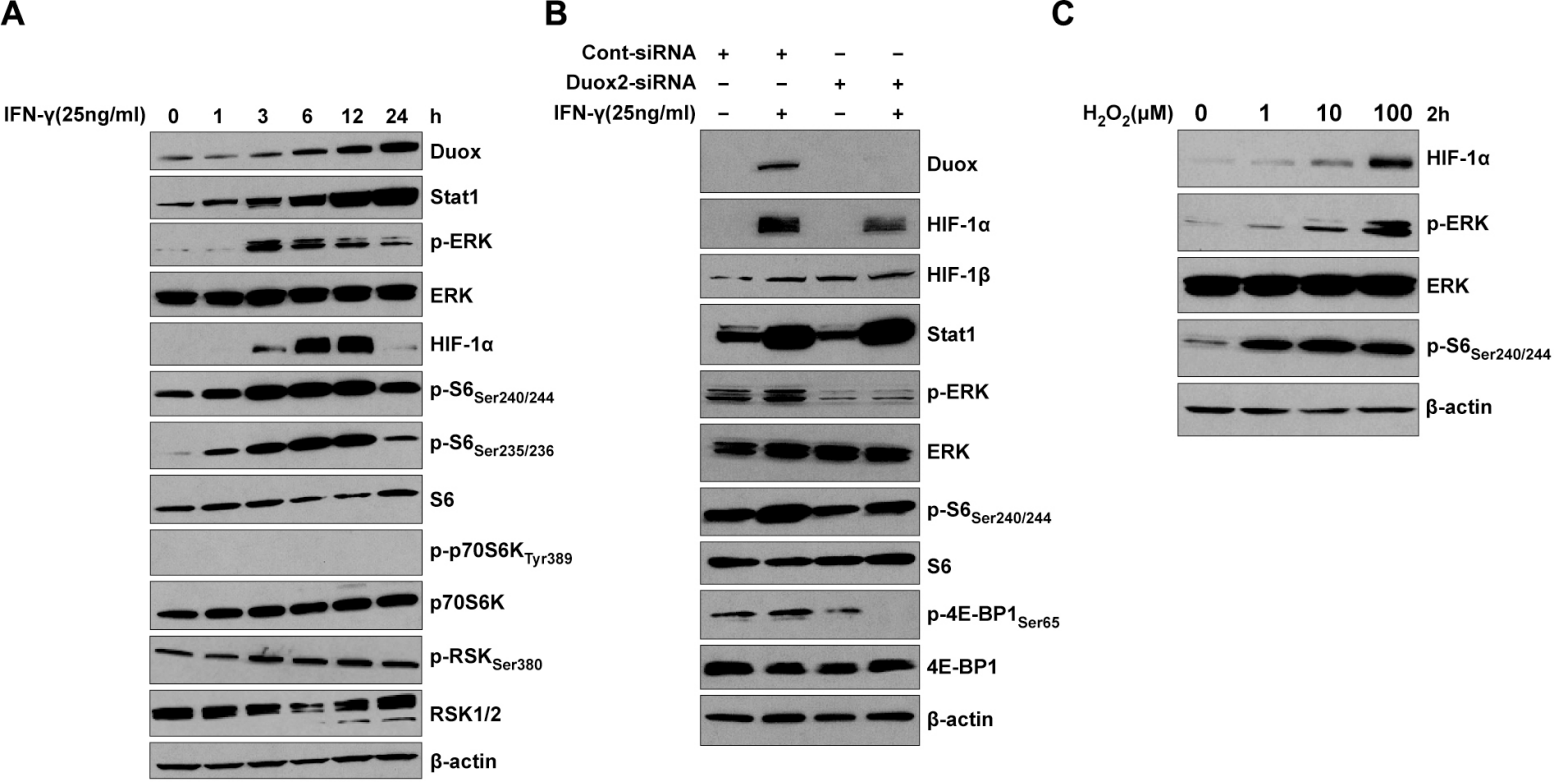
IFN-γ induces coordinate DUOX2 expression, ERK signaling, and HIF-1α synthesis in BxPC-3 cells in a fashion that resembles the effect of exogenous H_2_O_2_ (**A**) Western analysis of WCEs from BxPC-3 cells treated with IFN-γ for the indicated times. (**B**) Western analysis of WCEs from BxPC-3 cells transiently transfected with control or DUOX2-specific siRNA for 24 h, followed by IFN-γ treatment for 12 h. Expression of phosphorylated ERK in cells exposed to a control siRNA is likely related to transfection-induced stress. (**C**) Western analysis of WCEs from BxPC-3 cells treated with H_2_O_2_ at the indicated concentrations in serum-free medium for 2 h. In (A–C), β-actin served as a loading control. In all panels, the data are representative of at least three independent experiments.

Several important signaling pathways, including PI3K/Akt/FRAP, Raf/MEK/p42/p44 MAPK, and JAK/Stat, and their downstream transcription factor targets, such as HIF-1, Sp1, AP-1, and Stat3, play a critical role in the induction of VEGF-A by growth factors (IGF1, PDGF, EGF, and FGF), hormones (prostaglandin E2 and insulin), and cytokines (IL-1β, IL-6, and IL-8) in cancer cells [38, 39]. To examine the molecular mechanism of IFN-γ-induced VEGF-A expression in BxPC-3 pancreatic cancer cells, we monitored the kinetics of IFN-γ-induced ERK signaling (Figure 3A). Consistent with our previous report [12], IFN-γ strongly induced DUOX2 and Stat1 protein in a time-dependent manner in this cell line. In addition, HIF-1α expression was induced at 3 h and exhibited strong induction at 6 and 12 h, followed by a decrease in expression at 24 h. Notably, ERK phosphorylation was also induced beginning at 3 h, peaking at 3–6 h; and serine phosphorylation of the ribosomal protein and translation regulator S6 was induced at 1 h and peaked at 3–12 h, demonstrating an overlap in the kinetics of enhanced HIF-1α expression, and ERK and S6 activation. Moreover, IFN-γ modestly increased the activation of the ERK downstream target RSK, but did not activate Akt (data not shown) or alter the phosphorylation of a downstream target of Akt (p70S6K; Figure 3A). These results are consistent with reports that RAS/ERK signaling promotes site-specific S6 phosphorylation via RSK [40]. Although constitutively active Stat3 has been shown to directly regulate VEGF-A expression and associated angiogenesis and tumor growth in human pancreatic cancer [41], no effect of IFN-γ on the JAK/Stat3 signaling pathway was demonstrable in BxPC-3 cells, as indicated by a lack of Stat3 tyrosine phosphorylation (Supplementary Figure S1A). In contrast, IL-6 induced Stat3 activation (Supplementary Figure S1A) but did not affect DUOX2 or VEGF-A mRNA expression (Supplementary Figure S1B). Thus, IFN-γ-stimulated BxPC-3 cells exhibit induction of DUOX2, ERK activation, and HIF-1α protein synthesis, but not Akt or Stat3.

To confirm the role of IFN-γ-induced DUOX2 expression in ERK signaling and HIF-1α accumulation in BxPC-3 cells, we utilized DUOX2-specific small interfering RNA [siRNA]. As indicated in Figure 3B for IFN-γ-stimulated cells treated with a scrambled control siRNA, DUOX2, HIF-1α, and Stat1 protein expression, as well as ERK and S6 phosphorylation increased (similar to the findings presented in Figure 3A). Additionally, phosphorylation of 4E-BP1 was observed in parental BxPC-3 cells and those treated with control siRNA; 4E-BP1 phosphorylation prevents 4E-BP1-mediated inhibition of the translation initiation factor 4E, allowing assembly of the protein synthesis machinery. In IFN-γ-stimulated tumor cells exposed to a DUOX2-specific siRNA, however, in addition to a lack of DUOX2 expression and decreased HIF-1α expression, we observed diminished ERK and S6 activation (compare lane 4 to lane 2) as well as no phosphorylation of 4E-BP1. Taken together, these results demonstrate that IFN-γ-induced ERK signaling in BxPC-3 cells is DUOX2-dependent.

We then compared these DUOX2-mediated effects on signaling with those produced directly by H_2_O_2_ over a broad range of concentrations (Figure 3C). H_2_O_2_ triggered a concentration-dependent increase in HIF-1α expression as well as ERK and S6 phosphorylation. Activation of ERK as well as up-regulation of HIF-1α by H_2_O_2_ is consistent with previous findings in different cell lines [42–44]. The DUOX2-mediated effects on ERK signaling in IFN-γ-stimulated BxPC-3 cells that we observed are thus consistent with the function of DUOX2 as an H_2_O_2_-producing Nox.

### Transcriptional silencing of HIF-1α, Sp1, or Sp3 does not affect VEGF-A mRNA expression in IFN-γ-stimulated BxPC-3 cells

**Figure 4:**
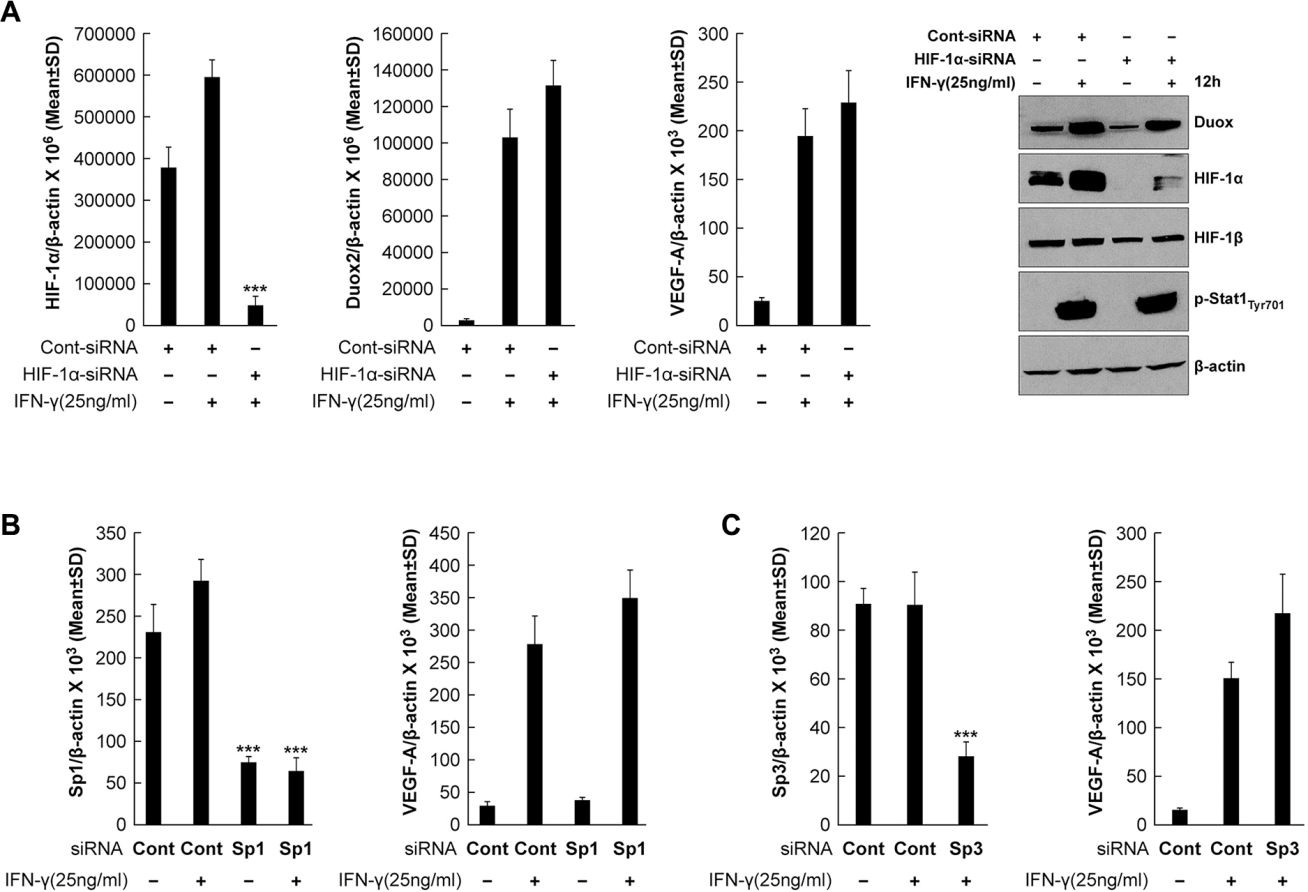
Transcriptional silencing of HIF-1α, Sp1, or Sp3 does not affect VEGF-A expression in IFN-γ-stimulated BxPC-3 cells (A) Quantitative PCR analysis of HIF-1α (left), DUOX2 (middle), and VEGF-A (right) expression and Western analysis (far right) of BxPC-3 cells transiently transfected with control or HIF-1α-specific siRNA for 24 h, followed by IFN-γ treatment for 12 h. β-actin served as a loading control. ***P < 0.001 vs. IFN-γ-stimulated, control siRNA-transfected cells. (B) Sp1 (left) and VEGF-A (right) expression in BxPC-3 cells transiently transfected with control or Sp1-specific siRNA for 24 h, followed by IFN-γ treatment for 12 h, as determined by quantitative PCR. ***P < 0.001 vs. IFN-γ-stimulated, control siRNA-treated cells. (C) Sp3 (left) and VEGF-A (right) expression in BxPC-3 cells transiently transfected with control or Sp3-specific siRNA for 24 h, followed by IFN-γ treatment for 12 h, as determined by quantitative PCR. ***P < 0.001 vs. IFN-γ-stimulated, control siRNA-treated cells. The data are expressed as the mean ± SD of at least three independent experiments (A, top; B–C) or are representative of at least three independent experiments (A, bottom).

We next sought to determine whether enhanced HIF-1α protein synthesis is responsible for increasing VEGF-A expression in IFN-γ-stimulated BxPC-3 cells. To do so, we exposed BxPC-3 cells to HIF-1α-specific siRNA prior to IFN-γ stimulation; this resulted in significant suppression of HIF-1α mRNA expression (*P* < 0.001 vs. IFN-γ-stimulated, control siRNA-transfected cells) but had no effect on DUOX2 or VEGF-A mRNA levels (Figure 4A). Similarly, HIF-1α silencing nearly eliminated HIF-1α protein expression but had no effect on DUOX protein (Figure 4A). To investigate whether the transcription factors Sp1 and Sp3, implicated previously in VEGF-A regulation in pancreatic cancer [45], mediate IFN-γ-induced VEGF-A expression in BxPC-3 cells, Sp1 and Sp3 were each silenced using siRNA. However, as shown in Figure 4B and 4C, despite effective knockdown of these transcription factors (*P* < 0.001 vs. IFN-γ-stimulated, control siRNA-transfected cells), VEGF-A expression was not significantly altered. In sum, neither HIF-1α, nor Sp1, nor Sp3 appear to be primarily responsible for the IFN-γ-mediated induction of VEGF-A in BxPC-3 cells.

### IFN-γ increases DUOX2 and VEGF-A expression and JunB/c-Jun signaling in BxPC-3 cells

**Figure 5:**
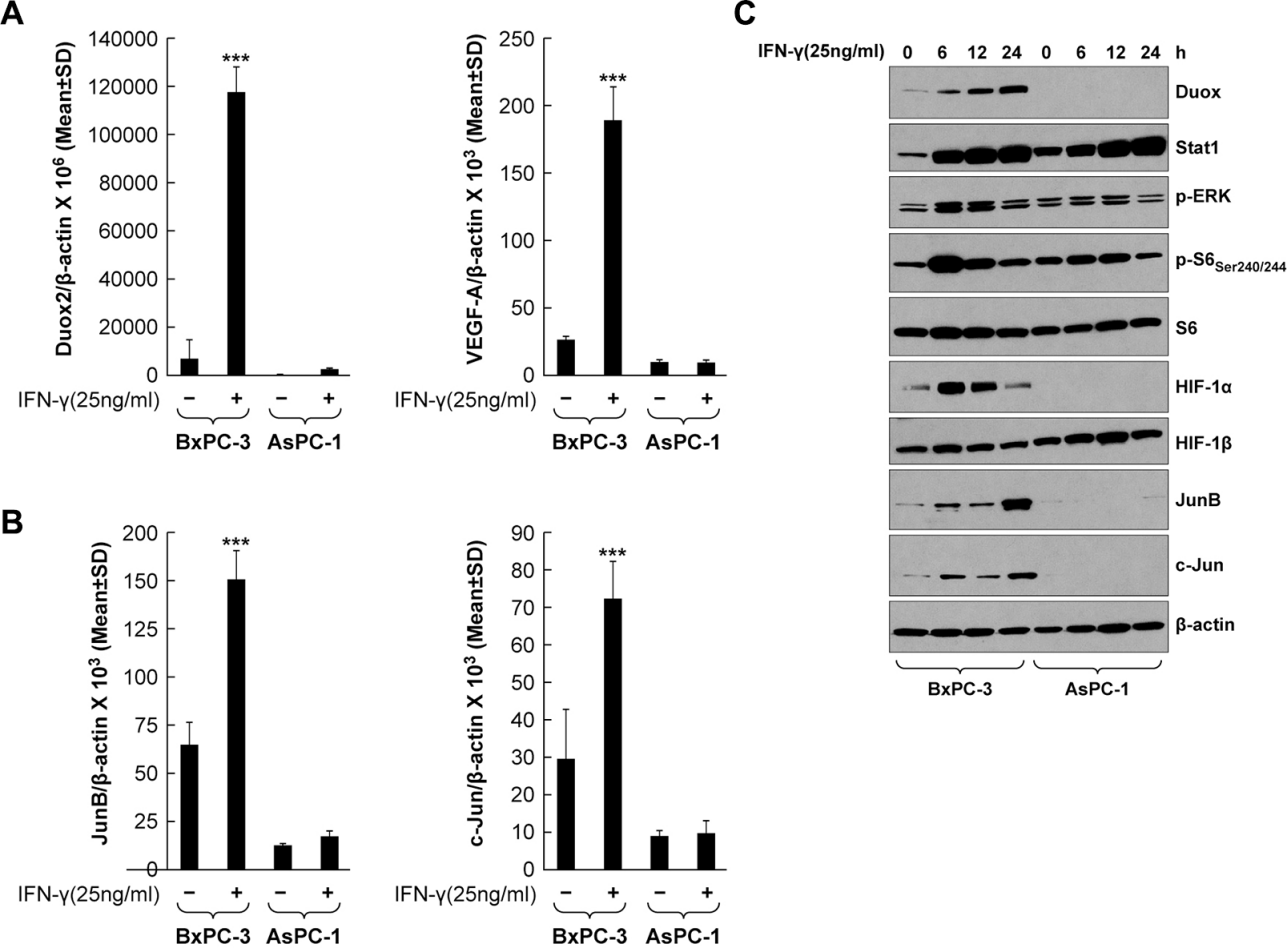
IFN-γ induces coordinate DUOX2 and VEGF-A expression and JunB/c-Jun activation in BxPC-3 cells (**A**) DUOX2 (left) and VEGF-A (right) expression in BxPC-3 and AsPC-1 cells with or without IFN-γ treatment for 24 h, as determined by quantitative RT-PCR. ***P < 0.001 vs. solvent-treated cells. (**B**) The same material as in A was analyzed for JunB (left) and c-Jun (right) expression. ***P < 0.001 vs. solvent-treated cells. (**C**) Western analysis of WCEs from BxPC-3 and AsPC-1 cells treated with IFN-γ for the indicated times. β-actin served as a loading control. The data are expressed as the mean ± SD of at least three independent experiments (A–B) or are representative of at least three independent experiments (C).

Transcription factors of the AP-1 family, including c-Jun and JunB, have been reported to participate in hypoxia- and hypoglycemia-mediated induction of VEGF-A. For example, c-Jun and JunB are activated by way of PKC and ERK signaling, respectively, in hypoglycemic mouse embryonic fibroblasts, resulting in VEGF-A up-regulation [46]. Therefore, we analyzed the role of these transcription factors in modulating VEGF-A expression in the BxPC-3 line, and compared the results with experiments performed using AsPC-1 cells (that are only weakly responsive to IFN-γ stimulation). Following IFN-γ treatment, the BxPC-3 line exhibited significantly up-regulated expression of DUOX2 and VEGF-A (*P* < 0.001, Figure 5A), as well as JunB and c-Jun (2- to 2.5-fold increases, *P* < 0.001, Figure 5B). Western analysis (Figure 5C) confirmed the quantitative RT-PCR results, demonstrating that JunB and c-Jun protein up-regulation continued for the duration of IFN-γ stimulation in BxPC-3 cells, but not in AsPC-1 cells. Increased DUOX2 and HIF-1α protein expression as well as ERK and S6 phosphorylation were observed concurrently in the IFN-γ-stimulated BxPC-3 cells. Notably, there was a clear difference in the kinetics of HIF-1α and JunB/c-Jun induction in these cells; although both HIF-1α and JunB/c-Jun protein expression levels were increased following 6 h of IFN-γ exposure, HIF-1α expression gradually decreased from 12–24 h, whereas JunB/c-Jun expression remained approximately the same at 12 h and then increased further by 24 h. Similar findings were obtained when CFPAC-1 cells were studied, including: significant increases in DUOX2 and VEGF-A mRNA expression (*P* < 0.001 for DUOX2 and *P* < 0.01 for VEGF-A; Supplementary Figure S2A); time-dependent increase in DUOX protein expression following IFN-γ exposure (Supplementary Figure S2C); activation of ERK and S6 (Supplementary Figure S2C); and up-regulation of HIF-1α and JunB protein expression (Supplementary Figure S2C). However, c-Jun is constitutively active in CFPAC-1 cells. A kinetic analysis of JunB and c-Jun mRNA and protein expression revealed that both transcriptional up-regulation and enhanced protein synthesis are responsible for the late accumulation of these two transcription factors in IFN-γ-stimulated BxPC-3 cells (data not shown). We therefore conclude that JunB/c-Jun signaling may play a role in IFN-γ-mediated induction of VEGF-A expression in BxPC-3 cells.

### IFN-γ-induced VEGF-A mRNA and protein expression is DUOX2-, H_2_O_2_-, and ERK-dependent in BxPC-3 cells

**Figure 6:**
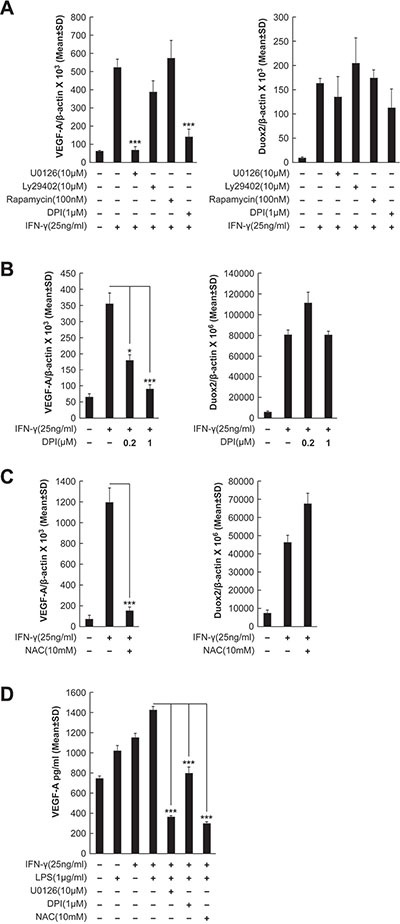
IFN-γ-induced VEGF-A mRNA and protein expression is DUOX2-, H_2_O_2_-, and ERK-dependent in BxPC-3 cells (**A**) VEGF-A (left) and DUOX2 (right) expression in BxPC-3 cells treated with the indicated inhibitors for 30 min, followed by IFN-γ treatment for 24 h, as determined by quantitative RT-PCR. U0126, Ly29402, rapamycin, and DPI inhibit MEK, PI3K, FRAP/mTOR, and flavoprotein dehydrogenases (including Noxs), respectively. ***P < 0.001 vs. IFN-γ-stimulated, non-inhibitor-treated cells. (**B**) VEGF-A (left) and DUOX2 (right) expression in BxPC-3 cells pretreated with DPI at the indicated concentrations, followed by treatment with IFN-γ for 24 h, as determined by quantitative RT-PCR. *P < 0.05, ***P < 0.001 vs. IFN-γ-stimulated cells without DPI treatment. (**C**) VEGF-A (left) and DUOX2 (right) expression in BxPC-3 cells pretreated with NAC for 30 min, followed by treatment with IFN-γ for 24 h, as determined by quantitative RT-PCR. ***P < 0.001 vs. IFN-γ-stimulated, non-NAC-treated cells. (**D**) ELISA analysis of VEGF-A protein expression in BxPC-3 cells pretreated with the indicated inhibitors for 30 min, followed by treatment with IFN-γ and/or LPS for 24 h. ***P < 0.001 vs. cells stimulated with IFN-γ and LPS alone. In all panels, the data are expressed as the mean ± SD of at least three independent experiments.

To investigate the signaling pathways involved in IFN-γ-mediated induction of VEGF-A expression in our BxPC-3 model further, tumor cells were exposed to the MEK inhibitor U0126, the PI3K inhibitor Ly29402, the FRAP/mTOR inhibitor rapamycin, or the inhibitor of flavin dehydrogenases (including Noxs) diphenylene iodonium [DPI], followed by IFN-γ stimulation. As shown in Figure 6A, IFN-γ stimulation alone resulted in an ≈ 10-fold increase in VEGF-A expression, which was significantly attenuated by either U0126 or DPI treatment (*P* < 0.001 vs. IFN-γ-stimulated, non-inhibitor-treated cells), but not by Ly29402 or rapamycin. In fact, VEGF-A levels after U0126 or DPI treatment were comparable to those in BxPC-3 cells without IFN-γ stimulation. Knockdown of either DUOX2 or DUOXA2, which are both necessary for the production of H_2_O_2_, also significantly decreased VEGF-A expression in IFN-γ-stimulated BxPC-3 cells (*P* < 0.01 vs. IFN-γ-stimulated, control siRNA-treated cells), whereas DUOX1 knockdown did not (Supplementary Figure S3). In contrast, as expected, IFN-γ-stimulated DUOX2 expression was not significantly affected by any of the inhibitors studied (Figure 6A).

Because high concentrations of DPI (> 1 μM) can produce off-target effects on enzymes other than Noxs [47], we examined VEGF-A and DUOX2 expression following treatment either with 1 μM DPI, as before, or with 0.2 μM DPI (Figure 6B); in previous work, we reported that the lower concentration of DPI effectively suppressed IFN-γ-induced H_2_O_2_ production in BxPC-3 cells [12] without altering mitochondrial respiration [48]. In the present study, we found that not only the higher concentration but also the lower concentration of DPI produced a significant inhibitory effect on VEGF-A expression (*P* < 0.001 and *P* < 0.05, respectively, vs. IFN-γ-stimulated cells without DPI treatment), whereas no change in DUOX2 levels was observed (Figure 6B).

To further characterize the effect of inhibiting DUOX2-related H_2_O_2_ production on VEGF-A expression, we assessed the consequences of treatment with the multi-functional reduced thiol (and glutathione precursor) n-Acetyl-L-cysteine [NAC] (Figure 6C). In IFN-γ-stimulated BxPC-3 cells, NAC treatment was associated with a significant decrease in VEGF-A expression (*P* < 0.001 vs. IFN-γ-stimulated non-NAC-treated cells), but not with a notable change in DUOX2 expression, supporting a relationship between DUOX2-derived H_2_O_2_ and VEGF-A up-regulation on the transcriptional level. On the translational level, in dually IFN-γ- and LPS-treated BxPC-3 cells, we also found significantly decreased VEGF-A protein expression in response to DPI and NAC, as well as to U0126, as determined by ELISA (*P* < 0.001 in all cases vs. cells stimulated with IFN-γ and LPS alone; Figure 6D). Therefore, up-regulated VEGF-A expression appears to depend on DUOX2-derived H_2_O_2_ and ERK signaling in IFN-γ-stimulated BxPC-3 cells.

### H_2_O_2_ is responsible for the enhanced ERK activation as well as increased HIF-1α and JunB/c-Jun expression in IFN-γ-treated BxPC-3 cells

**Figure 7:**
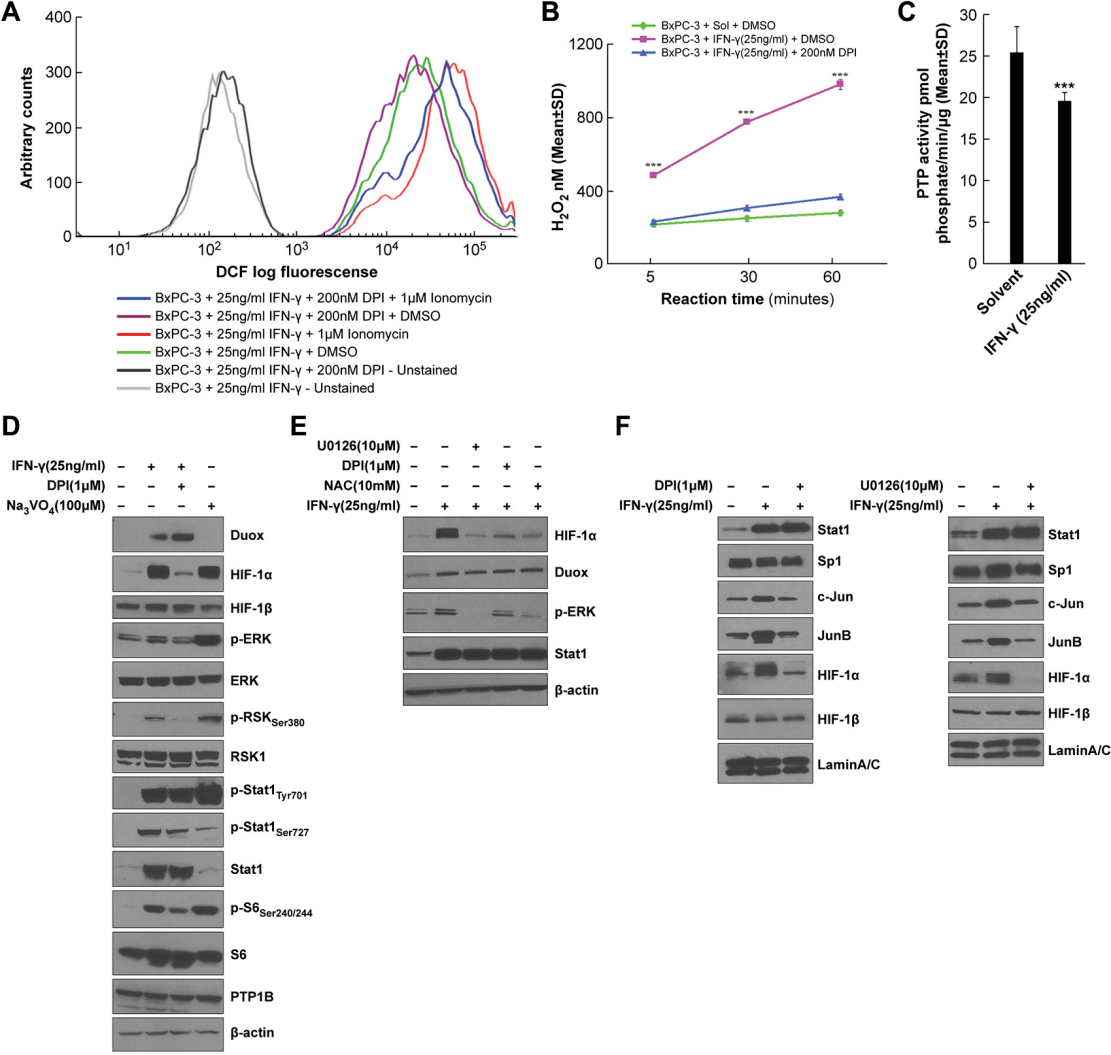
H_2_O_2_ is responsible for the enhanced ERK activation as well as HIF-1α and JunB/c-Jun expression in IFN-γ-treated BxPC-3 cells (**A**) Intracellular oxidant levels determined by analytical cytometry in BxPC-3 cells treated with IFN-γ for 24 h and with the flavoprotein dehydrogenase inhibitor DPI or DMSO for the last 2 h, followed by treatment with the dye CM-H2-DCF-DA for 30 min and with ionomycin or DMSO for the last 5 min. (**B**) Amplex Red® assay of extracellular H_2_O_2_ levels in BxPC-3 cells treated with IFN-γ for 24 h and with DPI or DMSO for the last 2 h, followed by exposure to ionomycin for the indicated times. H_2_O_2_ values were determined based on a standard curve of 0–2 μM H_2_O_2_. ***P < 0.001 vs. solvent- and DMSO-treated or IFN-γ- and DPI-treated cells. (**C**) PTP activity in BxPC-3 cells treated with or without IFN-γ for 24 h and with 1 μM ionomycin for the last 5 h. ***P < 0.001 vs. solvent-treated cells. (**D**) Western analysis of WCEs from BxPC-3 cells pretreated with DPI or DMSO for 30 min, followed by treatment with IFN-γ or the PTP inhibitor sodium orthovanadate [Na_3_VO_4_] for 12 h. (**E**) Western analysis of WCEs from BxPC-3 cells pretreated with the indicated inhibitors for 30 min, followed by treatment with IFN-γ for 12 h. U0126 is a MEK inhibitor, and NAC is a reduced thiol and glutathione precursor. F. Western analysis of nuclear extracts from BxPC-3 cells pretreated with DPI (left) or U0126 (right) for 30 min, followed by treatment with IFN-γ for 12 h. In D-E, β-actin served as the loading control, and in F, lamin A/C served as the loading control. The data are expressed as the mean ± SD of at least three independent experiments (B–C) or are representative of at least three independent experiments (A, D–F).

In light of the effects of exogenous H_2_O_2_ (Figure 3C) and DUOX-derived H_2_O_2_ (Figure 6A, 6C, and 6D) on VEGF-A expression in BxPC-3 cells, we next further examined intracellular oxidant and extracellular H_2_O_2_ production in IFN-γ-stimulated BxPC-3 cells. Figure 7A depicts the intracellular formation of a range of oxidants assessed using a chloromethyl derivative of the redox-sensitive dye 2′, 7′-dichlorodihydrofluorescein diacetate [H_2_-DCF-DA] known as CM-H_2_-DCF-DA [49]. In the absence of treatment with the redox-sensitive dye, with or without the Nox inhibitor DPI, only baseline cellular autofluorescence can be detected (shown as the two curves to the far left of Figure 7A), which is not a measure of ROS production. However, for BxPC-3 cells exposed to IFN-γ and stimulated with ionomycin, increased intracellular oxidant production was demonstrated by the conversion of H_2_-DCF-DA to the fluorescent species dichlorofluorescein [DCF]; shown in Figure 7A by the curve in red to the far right. DPI exposure decreased both IFN-γ-stimulated and IFN-γ- and ionomycin-stimulated intracellular oxidant levels, supporting a role for DUOX2 in intracellular oxidant production by BxPC-3 cells. Extracellular H_2_O_2_ generation was examined using Amplex Red^®^ under similar treatment conditions (all with ionomycin; Figure 7B) to the experiment reported in Figure 2F. BxPC-3 cells treated with IFN-γ produced significantly more H_2_O_2_ over time compared to cells not incubated with IFN-γ (*P* < 0.001 at all times); and DPI treatment decreased DUOX2-mediated H_2_O_2_ release by the IFN-γ-stimulated tumor cells to near-basal levels. CFPAC-1 cells exposed to IFN-γ and LPS and treated with ionomycin exhibited a similar increase in extracellular H_2_O_2_ production (Supplementary Figure S2B). We also found that HEK293 cells stably overexpressing human DUOX2 and DUOXA2 demonstrated high extracellular H_2_O_2_ production in parallel with ERK activation when treated with phorbol myristate acetate [PMA] and ionomycin (Supplementary Figure S4A, S4B, and S4C). These effects, which are independent of IFN-γ, were greater when DUOX2 and DUOXA2, rather than DUOX2 alone, were concomitantly expressed, and are consistent with the dependence of DUOX2 oxidase function on DUOXA2. Taken together, these results suggest that IFN-γ stimulates both intracellular oxidant formation in and extracellular H_2_O_2_ release from BxPC-3 and CFPAC-1 cells that are mediated by DUOX2.

H_2_O_2_ has been demonstrated to oxidize redox-sensitive cysteine residues in the catalytic pocket of PTPs, resulting in enzyme deactivation [50]. Furthermore, ERK activity is regulated in large measure by tyrosine phosphorylation, which is counteracted by the activity of PTPs. We found that ERK signaling is up-regulated in IFN-γ-stimulated cells in concert with increased DUOX2 expression and H_2_O_2_ production (Figure 3A and 3C); therefore, we investigated whether IFN-γ treatment might trigger sustained ERK activation by decreasing PTP activity. BxPC-3 cells exposed to IFN-γ followed by ionomycin demonstrated significantly decreased PTP activity compared to cells treated with solvent plus ionomycin (*P* < 0.001, Figure 7C). These results suggest that IFN-γ-mediated stimulation of H_2_O_2_ production by DUOX2 may enhance ERK activation, at least in part, by PTP inhibition. To characterize the effects of PTP inhibition on ERK signaling and HIF-1α accumulation further, we examined BxPC-3 cells treated with DPI plus IFN-γ or with the PTP inhibitor sodium orthovanadate (Figure 7D and 7E). In these experiments, DPI substantially decreased the levels of HIF-1α protein and phosphorylated ERK, RSK, S6, and Stat1 induced by IFN-γ. Sodium orthovanadate produced an opposite effect, increasing phosphorylation levels and triggering HIF-1α accumulation despite a lack of IFN-γ-stimulated DUOX2 up-regulation. These results suggest that PTP inhibition *per se* is sufficient to induce ERK-mediated signaling and a subsequent increase in HIF-1α protein expression in BxPC-3 cells. Thus, inhibition of PTP activity could play an important intermediary role linking enhanced DUOX2 activity and ERK-mediated HIF-1α accumulation.

To explore the connection between H_2_O_2_ production, ERK phosphorylation, and HIF-1α expression further, we revisited the effects of the MEK inhibitor U0126, the Nox inhibitor DPI, and the reduced thiol and glutathione precursor NAC on IFN-γ-stimulated BxPC-3 cells (Figure 7E). All three of these compounds decreased HIF-1α expression as well as levels of phosphorylated ERK in IFN-γ-treated cells but had no effect on DUOX protein levels, consistent with the involvement of DUOX2-derived H_2_O_2_ release and ERK activation in HIF-1α accumulation in BxPC-3 cells.

Finally, nuclear extracts from BxPC-3 cells were used to characterize the nuclear distribution of the studied transcription factors following IFN-γ stimulation. As shown in Figure 7F, both DPI and U0126 reduced the nuclear accumulation of JunB, c-Jun, and HIF-1α, but not Stat1 (which is DUOX2-independent) or Sp1 (which is constitutively expressed). Together with earlier results, these data suggest that IFN-γ-induced DUOX2-derived H_2_O_2_ deactivates PTP activity, leading, in part, to the activation of ERK signaling components and enhanced nuclear localization of the transcription factors HIF-1α, JunB, and c-Jun.

### DUOX expression is increased in both human PanINs and PDACs when compared to normal pancreatic tissues and concomitantly increased VEGF-A expression is observed in most PDACs

**Figure 8:**
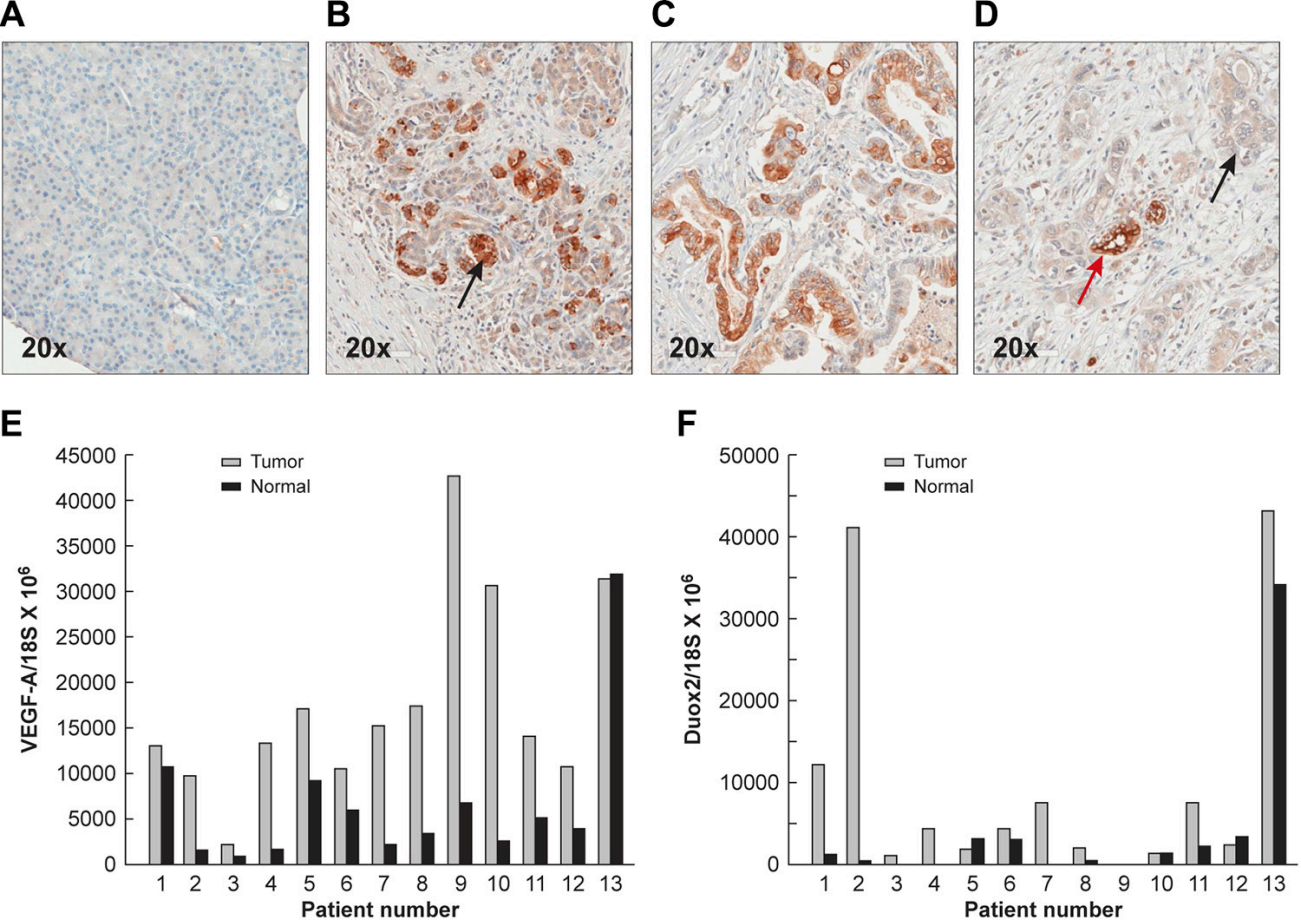
Expression of DUOX protein in the normal pancreas, PanINs, and PDACs demonstrated by immunohistochemistry, and increased mRNA expression levels of DUOX2 and VEGF-A in PDACs compared to adjacent, surgically-resected pancreatic tissues found to be tumor free (**A**) Normal human pancreas shown at 20 × power. Minimal to no staining for DUOX protein is seen by immunohistochemistry. (**B**) PanIN lesions demonstrate markedly increased cytoplasmic/membrane staining for DUOX protein (shown at the black arrow); however, the extent of these abnormal proliferative lesions may be limited in any particular section. (**C**) DUOX staining is frequent in grade 1 PDACs though often of lesser intensity than in PanIN lesions (Supplementary Table S1). (**D**) Grade 3 PDACs demonstrate weak DUOX staining in areas of atypical, invasive ducts (black arrow) and enhanced DUOX staining in less malignant ducts that reveal a greater degree of differentiation (red arrow). Relative VEGF-A (**E**) and DUOX2 F. expression levels normalized to 18S mRNA expression were determined in 13 pairs of human PDACs and adjacent tumor-free surgical samples using quantitative RT-PCR. The gray bars represent PDAC specimens; the black bars represent adjacent tissues from the same patient that had been found to be free of tumor following evaluation by an anatomic pathologist.

To evaluate whether DUOX2 might play a role in human pancreatic carcinogenesis, we studied the degree of DUOX expression in samples of human PanIN and PDAC as well as specimens of normal pancreatic tissue by immunohistochemistry [IHC] (Figure 8A–4D and Table 1, where “percent positive cells” refers to the proliferative lesions only, and not to the surrounding pancreas). We found that specimens of histologically normal pancreatic tissue did not display specific staining demonstrating expression of DUOX protein (Figure 8A). In contrast, 14/14 PanIN specimens revealed staining intensities of 2–3 in the majority of cells evaluated (*P* < 0.001 vs. normal pancreas; Table 1). PDAC cases revealed a significant increase in DUOX expression (as quantitated by both staining intensity and the percentage of positive cells) when compared to duct cells of the normal pancreas (Table 1); however, as the histological grade of the PDAC increased (consistent with more poorly differentiated tumors), both the intensity and the percentage of cells positive for DUOX protein decreased.

**Table 1:** Immunohistochemical analysis of DUOX expression in PanIN, PDAC, and normal pancreatic tissue specimens

Tissue Microarray Specimens	Staining Intensity (0–4)^a^						% Cells Positive^b^					
	0	1	2	3	4	Total	0	1	2	3	4	Total
Intraepithelial neoplasia (PanIN)	**0**	**0**	**2**	**12**	**0**	**14**	**1**	**1**	**2**	**8**	**2**	**14**
	0	0	14	86	0	%	8	7	14	57	14	%
Ductal adenocarcinoma [Grade 1]	**5**	**1**	**6**	**5**	**0**	**17**	**7**	**4**	**4**	**2**	**0**	**17**
	29	6	35	29	0	%	40	24	24	12	0	%
Ductal adenocarcinoma [Grade 1–2, 2]	**8**	**6**	**8**	**10**	**0**	**32**	**11**	**6**	**5**	**7**	**3**	**32**
	25	19	25	31	0	%	34	19	16	22	9	%
Ductal adenocarcinoma [Grade 2–3, 3]	**11**	**1**	**8**	**1**	**0**	**21**	**16**	**3**	**1**	**0**	**1**	**21**
	52	5	38	5	0	%	76	14	5	0	5	%
Normal pancreatic tissue adjacent to tumor	**9**	**0**	**0**	**0**	**0**	**9**	**9**	**0**	**0**	**0**	**0**	**9**
Normal pancreatic tissue	**9**	**0**	**0**	**0**	**0**	**9**	**9**	**0**	**0**	**0**	**0**	**9**

a cytoplasmic/membrane staining; 0 = none; 4 = strongest.

b 0 = 0 to < 10% of cells positive; 1 = 10–24% positive; 2 = 25–49%; 3 = 50–74%; 4 = 75–100%.

To establish the clinical relevance of our findings regarding the concerted DUOX2 and VEGF-A up-regulation by certain pro-inflammatory cytokines in PDAC cell lines, we studied 13 pairs of patient samples consisting of primary PDACs and adjacent, surgically-resected pancreatic tissues found to be free of tumor by light microscopy. All of these tissue specimens were subjected to RNA extraction and real-time RT-PCR to quantify the relative expression of both VEGF-A and DUOX2. As shown in Figure 8E, and consistent with prior investigations [24], VEGF-A mRNA expression in most patients with PDAC was up-regulated when compared to expression in adjacent pancreatic tissues not directly invaded by malignant cells. Among the 13 tumor/normal pairs, only one PDAC patient demonstrated less VEGF-A mRNA expression in tumor compared to the associated, uninvolved pancreas (patient 13). For the entire patient group, VEGF-A expression (mean ± SEM; ratio of VEGF-A mRNA to 18S mRNA × 10^6^) was 17,657 ± 3036 vs. 6,737 ± 2257 when PDAC samples were compared to adjacent normal tissues (*P* < 0.01). DUOX2 expression levels were increased in 9/13 pancreatic cancers when these samples were matched against adjacent pancreatic tissues that were without evidence of malignancy (Figure 8F). Overall, the expression of DUOX2 was significantly higher in PDACs than in the adjacent normal pancreas (mean ± SEM; 9945 ± 4090 vs. 3806 ± 2556; *P* < 0.04). These results are consistent with our observations in human PDAC cell lines *in vitro*; however, it is possible that the greater percentage of patients with VEGF-A up-regulation relative to DUOX2 up-regulation could be explained by a variety of mechanisms, including NOX2-related oxidant production by inflammatory cell infiltrates, or by other non-ROS related mechanisms that also play an important role in the regulation of VEGF-A expression in human tumors [51].

### Expression of VEGF-A, DUOX, and signaling proteins in BxPC-3 human pancreatic cancer xenografts from untreated mice is comparable to expression in BxPC-3 cells exposed to IFN-γ for 24 h *in vitro*

**Figure 9:**
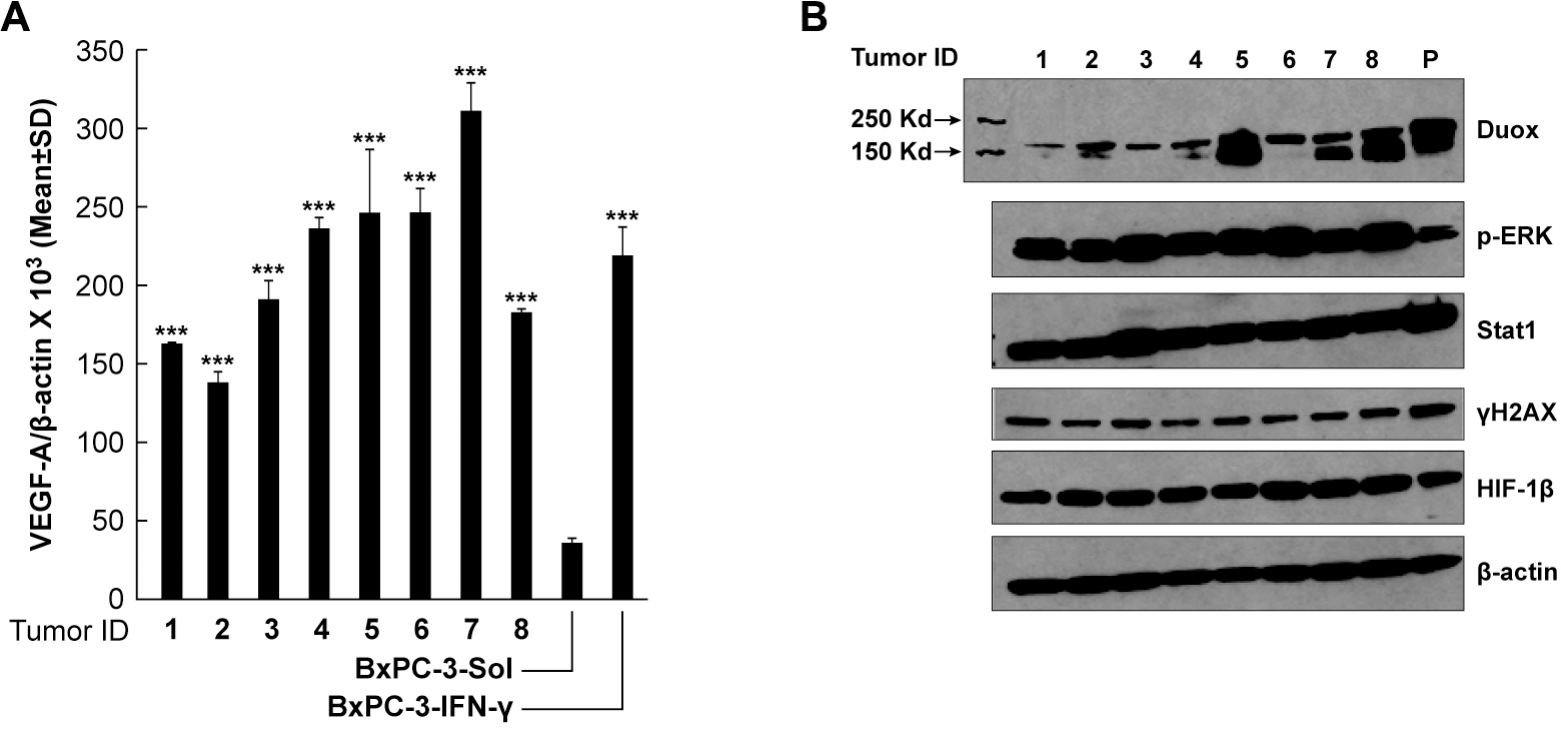
Expression of VEGF-A mRNA and DUOX and signaling proteins in BxPC-3 pancreatic cancer xenografts from untreated mice is comparable to expression in BxPC-3 cells exposed to IFN-γ for 24 h *in vitro* The fourth to sixth *in vitro* passages of cultured BxPC-3 cells were used to establish human tumor xenografts, as described in the Materials and Methods section; 300-mg tumor samples were split into quadrants, either for RNA extraction or for preparation of tumor lysates. (**A**) VEGF-A expression in BxPC-3 xenografts from individual mice was significantly increased when compared to VEGF-A expression in the BxPC-3 cell line immediately prior to inoculation, and was comparable to the expression level observed when the same cells were treated with 25 ng/ml IFN-γ for 24 h *in vitro*, as determined by quantitative RT-PCR. ***P < 0.001 for the comparison of VEGF-A expression in BxPC-3 xenograft tumors or BxPC-3 cells treated with 25 ng/ml IFN-γ for 24 h vs. solvent-treated cells. (**B**) Western analysis of cell lysates from the 8 individual BxPC-3 xenografts shown in panel (A) using specific antibodies, as indicated in the figure; “P” (in lane 9) denotes a WCE of BxPC-3 cells treated with 25 ng/ml IFN-γ for 24 h, shown as a positive control.

In a prior investigation, we demonstrated that BxPC-3 pancreatic cancer cells passaged for one cycle as xenografts in immunocompromised mice significantly up-regulated the expression of DUOX2 [11]. To extend those findings and determine whether the effects of DUOX2 on pro-angiogenic gene expression demonstrated above in cell lines occur *in vivo*, we evaluated the expression of VEGF-A as well as related signaling partners in eight BxPC-3 xenografts. As shown in Figure 9A, the expression of VEGF-A at the mRNA level increased 3- to 6-fold in BxPC-3 xenografts when levels in the tumor cell line immediately before implantation (bar labeled BxPC-3-Sol) were compared to those in solid tumors removed when they reached 300 mg in size (Tumor IDs 1–8). Moreover, VEGF-A mRNA expression in the xenografts (Tumor IDs 1–8), which were from animals not treated with exogenous cytokines, reached levels similar to those found *in vitro* following IFN-γ exposure (BxPC-3-IFN-γ; BxPC-3 cells exposed to 25 ng/ml IFN-γ for 24 h). Up-regulation of VEGF-A expression was accompanied by activation of ERK. The expression of the DNA damage marker γH2AX (Figure 9B) was also demonstrable in the xenografts and in BxPC-3 cells exposed to IFN-γ (labeled as ‘P’), in contrast to the low level of phosphorylated γH2AX that we previously reported for BxPC-3 cells exposed *in vitro* to solvent alone [11]. These data further support the close association between DUOX2 and VEGF-A expression in PDAC cells under pro-inflammatory conditions.

## DISCUSSION

We previously demonstrated that the pro-inflammatory cytokines IFN-γ and LPS increased DUOX2 expression, with concomitant H_2_O_2_ production and DNA damage, in human pancreatic cancer cell lines by activating signal transduction through both Stat1 and NF-κB [11]. Increased DUOX protein expression was also observed in patients with chronic pancreatitis (a pre-malignant condition) in proximity to areas of inflammatory cell infiltrates [11]. Our current experiments were performed to examine the consequences of cytokine-mediated DUOX2 up-regulation (and concomitant enhancement of H_2_O_2_ formation) on downstream effectors of malignant transformation and tumor progression. Demonstration in the current study that increased DUOX2-mediated H_2_O_2_ production in human pancreatic cancer cell lines was intimately associated with overexpression of VEGF-A and HIF-1α suggests that DUOX2 activity may contribute to the establishment of a pro-angiogenic milieu that adversely affects clinical outcome for patients with pancreatic cancer [23, 24].

Concentration-dependent enhancement of VEGF-A expression by IFN-γ in PDAC cells under normoxic conditions (Figure 1B) appears to be a consequence of H_2_O_2_-mediated ERK activation; the subsequent up-regulation of AP-1 expression by phosphorylated ERK could then target the VEGF-A promoter (Figure 5C). ERK activation was also observed in HEK293 cells that produce an abundance of H_2_O_2_ due to overexpression of DUOX2 and DUOXA2, rather than as a consequence of cytokine exposure (Supplementary Figure S4). In BxPC-3 cells, H_2_O_2_-related ERK activation might be explained, at least in part, by a significant decrease in the activity of tumor cell PTP activity coincident with active H_2_O_2_ formation (Figure 7C). In prior studies consistent with our observations, direct application of H_2_O_2_ has been demonstrated to increase VEGF mRNA stability in retinal pigment epithelial cells [52] and to activate ERK with consequent VEGF release from a human squamous cell carcinoma line [53]. Other investigators have suggested that Sp1, rather than AP-1, plays a critical role in regulating VEGF-A expression in gastric cancer cells [54] as well as in supporting the constitutive expression of VEGF in human pancreatic cancer [55]. The explanation for these differences may be found in the greater complexity of signal transduction along both MAPK and Akt pathways following growth factor binding to receptor tyrosine kinases [56], as opposed to the direct effects produced by DUOX2-related H_2_O_2_ production. It also seems possible that differences in tumor cell context (gastric versus pancreatic cancer) and the evaluation of baseline versus H_2_O_2_-induced transcription factor expression levels could help to explain why our results vary from those previously reported.

In addition to the adverse consequences of elevated VEGF-A levels in pancreatic cancer, abundant expression of HIF-1α contributes substantially to the pathophysiology of this disease [57, 58]. We found that under normoxic tissue culture conditions increased HIF-1α expression in PDAC cell lines was associated with DUOX2-related H_2_O_2_ production and ERK activation following exposure to IFN-γ, and that these results could be mimicked by the short-term exposure of PDAC cells to concentrations of exogenous H_2_O_2_ as low as 1 μM (Figure 3C). The concentration-dependent decrease in IFN-γ-stimulated HIF-1α expression produced by PEG-catalase also supports a central role for H_2_O_2_ in the modulation of HIF-1α in the studies reported here. H_2_O_2_-dependent up-regulation of HIF-1α protein expression (under both normoxic and hypoxic conditions) has been attributed previously to a variety of potential biochemical mechanisms. These include: altered regulation and inactivation of prolyl hydroxylases, in part through the oxidation of critical stores of Fe^2+^ [43]; diminished levels of intracellular reducing equivalents such as ascorbate, that would decrease degradation of HIF-1α protein; H_2_O_2_-related transcriptional activation of NF-κB with subsequent binding to the HIF-1α promoter [34]; and activation of components of either the MAPK or Akt pathways capable of increasing HIF-1α synthesis [59].

In the absence of data demonstrating an effect of DUOX2 up-regulation on HIF-1α mRNA levels (Figure 2A), and in view of our studies with cycloheximide and MG132 (Figure 2D and 2E) as well as the MAPK inhibitor U0126, the reduced thiol and glutathione precursor NAC, and the flavin dehydrogenase inhibitor DPI (Figure 7D–7F), it is reasonable to suggest that enhanced protein synthesis related to H_2_O_2_-dependent ERK activation is the most likely explanation for the increased levels of HIF-1α that we observed following cytokine exposure of PDAC cells. It is important to point out that we could not demonstrate, utilizing shRNA silencing, a direct, transcriptional effect of HIF-1α up-regulation on the expression of VEGF-A under normoxic conditions (Figure 4A). Thus, while VEGF-A expression and HIF-1α expression appear to be increased in a H_2_O_2_-dependent fashion in PDAC cells, we have not demonstrated that HIF-1α is directly upstream of VEGF-A in these experiments.

To understand the potential relevance of our studies conducted with PDAC cell lines to the clinical behavior of pancreatic cancer, we evaluated the expression of DUOX2 *in vivo*. Using an immunohistochemical approach, we found that DUOX protein was expressed substantially in PanIN patient specimens and in the preponderance of tissues from patients with frank PDAC (*P* < 0.01) for all PDAC grades and PanINs when compared to expression in normal pancreatic tissues (where DUOX expression was scant; Figure 8). Furthermore, DUOX expression appeared to decrease in degree and extent as the pancreatic tumors progressed from the pre-malignant, to the well-differentiated, and finally to the poorly differentiated stage (Table 1). In view of our previous results demonstrating marked expression of DUOX in patients with chronic pancreatitis, these data suggest that DUOX2 might be involved in the early phases of pancreatic carcinogenesis and cancer progression.

Our immunohistochemical observations were confirmed at the mRNA level (Figure 8E and 8F). Most of the 13 surgically-resected PDAC samples we evaluated expressed DUOX2 at a higher level in tumor than in adjacent, resected uninvolved pancreas (*P* < 0.04 for the group as a whole). VEGF-A expression was also significantly increased in the same set of tumors versus adjacent uninvolved pancreatic tissues from the same individuals. It should be noted, however, that by RT-PCR it was not possible to distinguish a potential contribution of endothelial or smooth muscle cell (versus tumor cell) expression of VEGF-A mRNA to our measurements. Furthermore, although the association of significantly increased DUOX2 and VEGF-A expression levels in these studies supports our cell line investigations, a causal relationship remains to be determined in view of the wide range of mechanisms, in both hypoxic and normoxic environments, potentially involved in the regulation of VEGF-A expression *in vivo* [51].

Finally, we demonstrated in human tumor xenografts that a single passage of BxPC-3 PDAC cells *in vivo* resulted in a significant up-regulation of VEGF-A mRNA (Figure 9A) and DUOX protein (Figure 9B). When compared to the parental BxPC-3 cells used to establish these xenografts (unexposed to IFN-γ; Figure 3A), the presence of ERK activation and the expression of the DNA damage marker γH2AX were notable across all of the xenograft specimens. These results suggest the hypothesis that tumor-associated macrophages and potentially other components of the tumor microenvironment known to produce pro-inflammatory cytokines or other immunologically-active species, such as IFN-γ [60, 61], could have significantly altered gene expression (DUOX2) and signal transduction pathways (ERK) in our pancreatic cancer xenografts.

**Figure 10:**
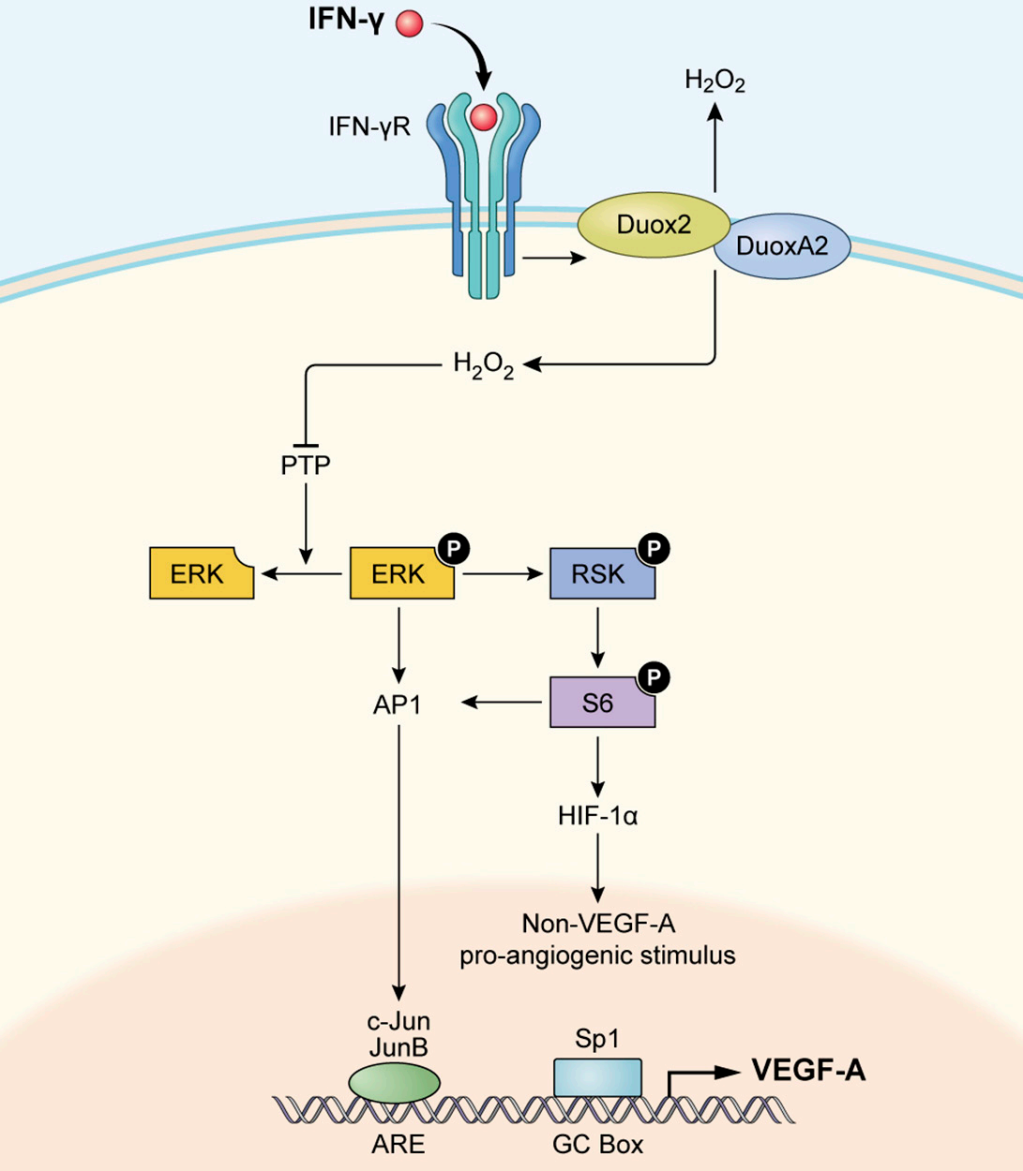
Model illustrating how IFN-γ-induced DUOX2 expression and H_2_O_2_ production stimulate HIF-1α, JunB, and c-Jun expression and subsequent VEGF-A up-regulation in BxPC-3 cells By engaging its cognate receptor (IFN-γR) on pancreatic cancer cells, IFN-γ up-regulates functional DUOX2/DUOXA2 expression that leads to enhanced H_2_O_2_ production and oxidative inactivation of PTPs. PTP inactivation could then contribute to ERK, RSK, and S6 phosphorylation, leading to accumulation of HIF-1α and subsequent pro-angiogenic signal transduction, as well as concomitantly increasing expression of AP-1 (consisting of JunB/c-Jun). Binding of c-Jun and JunB to its transcriptional response element (ARE, AP-1 response element) in the nucleus, along with binding of the constitutively expressed transcription factor Sp1 to the GC box, appears to stimulate VEGF-A expression in BxPC-3 cells.

In summary, as modeled in Figure 10, we suggest that increased expression of DUOX2/A2, under the influence of IFN-γ or following transfection of both DUOX2 and DUOXA2, leads to a significant increase in H_2_O_2_ production and consequent activation of ERK signaling, in part through the oxidative inactivation of PTPs. Activated ERK then supports a significant increase in HIF-1α synthesis and the translocation of AP-1 to the nucleus where it can stimulate the expression of VEGF-A. In this fashion, H_2_O_2_ produced in either normoxic or hypoxic areas of pancreatic tumors or pre-malignant lesions could serve as a potent angiogenic stimulus, enhancing a pro-inflammatory oxidant milieu that is conducive to the maintenance of genetic heterogeneity, tumor invasion, and therapeutic resistance [62].

## MATERIALS AND METHODS

### Reagents and antibodies

Recombinant human IFN-γ (cat# 285-IF0) and IL-6 (206-IL-010) were purchased from R&D Systems (Minneapolis, MN, USA). Actinomycin D (A9415), cycloheximide (C7968), DPI (D2926), MG132 (C2211), NAC (A7250), PEG-catalase (C4963), and sodium orthovanadate (S6508) were from Sigma-Aldrich (St. Louis, MO, USA). LPS (437625), Ly29402 (440202), rapamycin (553210), and U0126 (662005) were obtained from EMD Chemicals (Gibbstown, NJ, USA). Cobalt chloride was purchased from Acros Organics (7646-79-9; Geel, Belgium). PMA was from EMD Millipore (19–44; Temecula, CA, USA). DMSO was used as the solvent for all of the above reagents except for IFN-γ, IL-6, and LPS, which were dissolved in 0.1% BSA in PBS; NAC and cobalt chloride were dissolved in 1X PBS. H_2_O_2_ was from Calbiochem (386790; San Diego, CA, USA). Anti-human DUOX antibody, which reacts with both DUOX1 and DUOX2, was previously developed by Creative Biolabs (Port Jefferson Station, NY, USA) and characterized by our laboratory [12]. Anti-human HIF-1α antibody (610959) was from BD Transduction Laboratories (San Diego, CA, USA). Anti-human Stat1 (sc-346), Sp1 (sc-59), and HRP-tagged goat anti-rabbit IgG (sc-2054) and goat anti-mouse IgG (sc-2055) were from Santa Cruz Biotechnology (Santa Cruz, CA, USA). Antibodies against human HIF-1β (3718), c-Jun (9165), JunB (3746), lamin A/C (2032), ERK (9102), p-ERK (9101), p70S6K (2708), p-p70S6K_Thr389_ (9234), PTP1B (5311), RSK1 (8408), RSK1/2/3 (9355), p-RSK_Ser380_ (9335), S6 (2217), p-S6_Ser235/236_ (4856), p-S6_Ser240/244_ (2215), p-Stat1_Ser727_ (9177), p-Stat1_Tyr701_ (9167), Stat3 (8768), p-Stat3_Tyr705_ (9145), 4E-BP-1 (9644), and p-4E-BP-1_Ser65_ (9456) were purchased from Cell Signaling Technology (Beverly, MA, USA). Human β-actin (Hs99999903_m1), DUOX1 (Hs00213694_ml), DUOX2 (Hs00204187_m1), DUOXA2 (Hs01595310_g1), HIF-1a (Hs00153153_m1), c-Jun (Hs00277190_s1), JunB (Hs00357891_s1), Sp1 (Hs00916518_m1), Sp3 (Hs01595811_m1), and VEGF-A (Hs00900055_m1) primers were from Life Technologies (Carlsbad, CA, USA). Negative Control #1 (AM4635) and human DUOX2 (s27012), DUOXA2 (s53881), HIF-1α (s6539), JunB (s7663), c-Jun (s7658), Sp1 (s13319), and Sp3 (s13326) siRNAs were obtained from Applied Biosystems (Carlsbad, CA, USA).

### Cell culture

The human pancreatic cancer cell lines BxPC-3 (cat# CRL-1687), AsPC-1 (CRL-1682), CFPAC-1 (CRL-1918), and PANC-1 (CRL-1469) were obtained from the American Type Culture Collection (ATCC) (Manassas, VA, USA). Both BxPC-3 and AsPC-1 cells were cultured in RPMI 1640 medium (SH30255.01; HyClone, Logan, UT, USA) with 1.0% sodium pyruvate and 10% FBS (100–106; Gemini Bio Products, Sacramento, CA, USA). The CFPAC-1 cells were cultured in IMDM (30–20065; ATCC) with 10% FBS. Each cell line's identity was confirmed by the Genetic Resources Core Facility of Johns Hopkins University (Baltimore, MD, USA). Additionally, HEK293 cell lines that stably express the human *DUOX2* gene alone or with human *DUOXA2* were kindly provided by Dr. William M. Nauseef (University of Iowa, Iowa City, IA, USA) and were maintained as described [63]. To establish starvation conditions before each experiment, cells were cultured overnight in the same medium without FBS. Starvation conditions were used because DUOX2 induction by IFN-γ is stronger after serum starvation, as noted previously [11, 12]. In all cases, cells were cultured in a humidified incubator at 37°C in an atmosphere of 5% CO_2_ in air. We previously found that the basal levels of all Nox homologues (Nox1-5 and DUOX1-2) were negligible in BxPC-3, AsPC-1, and CFPAC-1 cells, as determined by real-time RT-PCR [11].

### Mice

All mice (female athymic nu/nu NCr) used in this study were obtained from the Animal Production Area of the Frederick National Laboratory for Cancer Research (Frederick, MD, USA). NCI-Frederick is accredited by the Association for Assessment and Accreditation of Laboratory Animal Care International and follows the Public Health Service Policy on Humane Care and Use of Laboratory Animals. Humane animal care was also provided according to the protocols described in the Guide for the Care and Use of Laboratory Animals (Eighth Edition) of the National Research Council and the U.S. Government Principles for the Utilization and Care of Vertebrate Animals Used in Testing, Research, and Training (1985).

### Quantitative real-time RT-PCR

Total RNA was extracted from cells using the RNeasy Mini Kit (cat# 74104; Qiagen, Valencia, CA, USA) following the manufacturer's instructions. Two micrograms of total RNA were then used for cDNA synthesis, along with SuperScript II Reverse Transcriptase (18080–044) and random primers (48190–011) (both from Life Technologies), in a 20 μl reaction. The RT-PCR conditions consisted of cycles of 25°C for 10 min, 42°C for 50 min, and 75°C for 10 min. The RT-PCR products were diluted to 100 μl with diethylpyrocarbonate-treated H_2_O, and real-time RT-PCR was conducted in 384-well plates in a 20 μl volume consisting of 2 μl of diluted cDNA, 1 μl of the appropriate primers, 7 μl of H_2_O, and 10 μl of 2× TaqMan Universal PCR Master Mix (4364340; Life Technologies). The PCR reaction was performed using the default cycling conditions (50°C for 2 min, 95°C for 10 min, and 40 cycles of 95°C for 15 sec and 60°C for 10 min) with the ABI Prism 7900HT Sequence Detection System (Applied Biosystems). Triplicate samples were used for the real-time RT-PCR, and the mean values were calculated. The data in all Figure represent three independent experiments. Relative gene expression was calculated from the ratio of the target gene expression to the internal reference gene (β-actin) expression based on the Ct values.

### Western analysis

To prepare extracts, cells were lysed in 1× RIPA Lysis Buffer (cat# 20–188; EMD Millipore) supplemented with a phosphatase inhibitor tablet (04-906-837001) and a protease inhibitor tablet (11-836-153001) (both from Roche, Mannheim, Germany) to generate whole-cell extracts [WCEs]. Nuclear extracts were additionally prepared using the NE-PER Nuclear and Cytoplasmic Extraction Kit (78833; Thermo Scientific, Rockford, IL, USA). The protein concentrations of both types of extracts were measured using the BCA Protein Assay Kit (23227; Pierce, Rockford, IL, USA). The extracts were then combined with an equal volume of 2× SDS Protein Gel Loading Solution (351-082-661; Quality Biological, Gaithersburg, MD, USA). Next, unless otherwise indicated, 50 μg of WCE or 20 μg of nuclear extract was electrophoretically separated on a 4–20% Tris-glycine gel (EC6028; Life Technologies) and transferred to nitrocellulose membranes using an iBlot Transfer Stack (IB 3010-01; Life Technologies). The membranes were blocked in 5% nonfat milk in 1× TBST (TBS with 0.1% Tween 20) buffer for 1 h at room temperature and then incubated overnight with the indicated primary antibodies in TBST. After three washes in TBST, the membranes were incubated with the appropriate HRP-conjugated secondary antibodies for 1 h at room temperature on a shaker. SuperSignal West Pico Luminol/Enhancer Solution (1856136; Thermo Scientific) was then applied to visualize the proteins of interest. To assess DUOX protein expression in particular, the WCEs were combined with an equal volume of 2× SDS loading buffer without boiling, whereas for other proteins, the mixture was boiled for 5 min. This protocol was adopted because DUOX2 in WCEs forms an aggregate upon boiling, leading to reduced electrophoretic mobility, as we have previously noted [12].

### ELISA

An ELISA for the quantitative determination of human VEGF concentrations in cell culture supernatants was performed using a Human VEGF Quantikine ELISA Kit (cat# DVE00; R&D Systems) following the manufacturer's instructions. For this purpose, a total of 1 × 10^6^ cells in complete medium were seeded in a 60 mm tissue culture dish. After an overnight incubation, the medium was changed to serum-free medium; the cells were starved for 30 min and then treated with various cytokines/chemicals for 24 h, as indicated. The supernatant was collected subsequently, and the appropriate amount of supernatant then used for the ELISA.

### Analytical cytometry

To measure intracellular oxidant levels, a total of 1 × 10^6^ cells per sample were suspended in 0.5 ml of Krebs buffer containing 7.5 μM CM-H_2_-DCF-DA (cat# C6827; Life Technologies), a redox-sensitive dye, and incubated in the dark for 30 min at 37°C. Following 25 min of incubation, either ionomycin (407952; EMD Chemicals) (final concentration of 1 μM) or the same volume of DMSO was added to the cell suspension for the last 5 min. Cells were then harvested and resuspended in medium lacking CM-H_2_-DCF-DA, and fluorescence was measured using a FACSAria flow cytometer (BD Biosciences, San Jose, CA, USA) in the FL-1 channel and analyzed with FlowJo^®^ software (Tree Star, Ashland, OR, USA).

### Amplex Red^®^ assay

The Amplex Red^®^ Hydrogen Peroxide/Peroxidase Assay Kit (cat# A22188; Life Technologies) was employed to detect extracellular H_2_O_2_ release. Cells were washed twice with 1× PBS, trypsinized, and counted. Next, for BxPC-3 and CFPAC-1 cells, 20 μl of cell suspension containing 2 × 10^4^ live cells in 1× Krebs-Ringer phosphate glucose [KRPG] buffer was mixed with 100 μl of a solution containing 50 μM Amplex Red^®^ and 0.1 U/ml HRP in KRPG buffer with 1 μM ionomycin and incubated at 37°C for the indicated times. For HEK293-DUOX2 and 2A2 cells, 1.5 × 10^4^ cells in 20 μl of 1× KRPG buffer were mixed with 50 μM Amplex Red^®^ and 0.25 U/ml HRP in KRPG buffer with or without 1 μM ionomycin or PMA/ionomycin (400 nM PMA plus 1 μM ionomycin). The fluorescence of the oxidized 10-acetyl-3,7-dihydroxyphenoxazine was then measured at excitation and emission wavelengths of 530 nm and 590 nm, respectively, using a SpectraMax Multi-Mode Microplate Reader (Molecular Devices, Sunnyvale, CA, USA), and the amount of extracellular H_2_O_2_ was determined using a standard curve from 0–2 μM H_2_O_2_. Each value in the Figure is the mean value for triplicate or quadruplicate samples.

### PTP assay

A PTP Assay Kit (cat# 17-125; EMD Millipore) was used to measure PTP activity. Cells were first scraped into lysis buffer containing 20 mM HCl, 2 mM EDTA, and 2 mM EGTA (pH 7.0) supplemented with a protease inhibitor cocktail (78425; Pierce), followed by sonication and centrifugation at 2000 × *g* for 5 min. The protein concentration in the supernatant was measured with a BCA Protein Assay Kit; 1 μg of protein was used per well. PTP activity was then determined according to the manufacturer's instructions. Briefly, in 96-well half-area plates, the indicated amount of protein and 200 μM peptide (RRLIEDAEpYAARG) were added in a 25 μl total volume. After incubation for 15 min, the enzyme reaction was terminated with 100 μl of malachite green solution from the kit. An additional 15 min were allowed for color development, and the absorbance was measured at 650 nm with a SpectraMax M5 plate reader. Enzyme activity was then calculated based on the amount of released phosphate in pmol phosphate/min/μg protein based on a phosphate standard curve.

### Tumor xenografts

For mouse inoculation, BxPC-3 cells were used at the 4^th^ to 6^th^ *in vitro* passage of cryopreserved cell stocks. In particular, the cells (1 × 10^7^ cells/0.1 ml/injection) were subcutaneously inoculated bilaterally into mice (*n* = 20 mice); ten mice each were randomly allocated to 300 mg and 500 mg tumor harvest groups on the day of inoculation. Upon reaching the target size, the tumors were resected, cut into quadrants, flash frozen in pre-chilled cryovials by submersion in liquid nitrogen, and stored in a –70°C freezer until processing. For the current experiments, the 300 mg tumor harvest group inoculated with BxPC-3 cells was studied; eight of these ten mice produced non-necrotic tumors suitable for further evaluation.

### Immunohistochemical determination of DUOX expression in tumor tissue microarrays of human PanINs and PDACs as well as normal pancreas

Three tumor tissue microarrays [TMAs] were obtained from US Biomax (cat. # PA1001, BIC14011, PA485; Rockville, MD, USA). The TMAs contained human pancreatic specimens ranging from normal, through intraepithelial neoplasm, to grade 3 ductal adenocarcinomas. For immunohistochemical studies, slides were deparaffinized and antigen retrieval was performed at 95°C for 20 min (DAKO Target Retrieval Solution [cat. # S1699; DAKO, Carpinteria, CA, USA]). After cooling for 20 min, slides were rinsed in distilled water, transferred to TBST buffer (cat. # S3006; DAKO) for 5 min, and then incubated in peroxidase blocking solution (3% H_2_O_2_) for 10 min at room temperature. Anti-DUOX antibody, diluted to 1:250 (in DAKO Antibody Diluent; cat. # S0809) was applied for 60 min at room temperature. Slides were subsequently rinsed in TBST, and secondary antibody (DAKO EnVision+ Dual Link System-HRP [cat. # K4063], Ready-To-Use) was applied for 30 min at room temperature. A TBST rinse was performed 3 times for 2 min each before applying 3,3′-diaminobenzidine for 10 min for visualization. Slides received a final rinse in TBST before being counterstained with hematoxylin. Positive controls (normal human colon; cell pellet) and a negative control reagent (mouse IgG1 isotype [cat. # 550878; BD Biosciences] in DAKO diluent [cat. # S0809], 10 μg/ml) were used for 60 min each and run at the same time. TMAs were then scanned into ScanScope CS (Aperio, Buffalo Grove, IL, USA) at × 40. Images were evaluated and graded by a board-certified veterinary pathologist for staining intensity and the percentage of positive cells.

### mRNA expression of DUOX2 and VEGF-A in human pancreatic cancers and adjacent non-malignant pancreatic tissues

Primary pancreatic cancer and adjacent non-malignant pancreatic tissue samples were acquired from the National Cancer Institute-sponsored Cooperative Human Tissue Network (Eastern, Mid-Western, and Mid-Atlantic Divisions) in compliance with the Office of Human Subjects Research at the National Institutes of Health, Bethesda, MD. Specimens were selected without regard to age, race/ancestry, or sex and were obtained from patients who had not received chemotherapy or radiation treatment prior to surgical intervention. Tumors were preserved by snap-freezing in liquid nitrogen within 60 min of surgery. For analysis, tissues ranging in size from 200–750 mg were homogenized on ice, and RNA was isolated utilizing the RNeasy Plus Universal Mini Kit (cat# 73404; Qiagen) according to the manufacturer's protocol. Two micrograms of total RNA isolated from each specimen was utilized for cDNA synthesis in a 20-μl reaction system with the following cycles: 25°C for 5 min, 42°C for 50 min, and 75°C for 5 min. After the reaction was complete, the RT-PCR products were diluted with H_2_O to 100 μl prior to quantitative real-time PCR. For the analysis of surgical specimens of human tumors and adjacent normal tissues, 18S was used as the housekeeping control gene.

### Statistical analysis

Statistical differences between the mean values of samples were assessed using the two-tailed Student's *t* test, the two-tailed Wilcoxon matched-pairs signed-rank test, or the two-tailed Fisher's exact test. Statistical significance was defined as *P < 0.05, **P < 0.01, and ***P < 0.001.

## SUPPLEMENTARY MATERIALS FIGURES



## Abbreviations

DUOX2: dual oxidase 2
VEGF: vascular endothelial growth factor
HIF-1α: hypoxia-inducible factor-1α
PDAC: pancreatic ductal adenocarcinoma
ROS: reactive oxygen species
PTPs: protein tyrosine phosphatases
IFN-γ: interferon-γ
Nox: NADPH oxidase
LPS: lipopolysaccharide
ERK: extracellular signal-related kinase
Sp1: stimulatory protein 1
AP-1: activator protein 1
PEG-catalase: polyethylene-modified catalase
PanIN: pancreatic intraepithelial neoplasia
DPI: diphenylene iodonium
siRNA: small interfering RNA
PMA: phorbol myristate acetate
H2-DCF-DA: 2′,7′-dichlorodihydrofluorescein diacetate
WCE: whole cell extract
NAC: n-Acetyl-L-cysteine
KRPG: Krebs-Ringer phosphate glucose
TMA: tissue microarray
