# Supplementary material for: Dual oxidase 2 and pancreatic adenocarcinoma: IFN-γ-mediated dual oxidase 2 overexpression results in H2O2-induced, ERK-associated up-regulation of HIF-1α and VEGF-A
Source: Oncotarget. 2016 Sep 15;7(42):68412–33. doi: 10.18632/oncotarget.12032 (PMC5340089; doi:10.18632/oncotarget.12032)
Supplement: Supplementary file 1 [file oncotarget-07-68412-s001.pdf]

Dual oxidase 2 and pancreatic adenocarcinoma: IFN-γ-mediated dual oxidase 2 overexpression results in H_2O_2-induced, ERK-associated up-regulation of HIF-1α and VEGF-A

Supplementary Materials

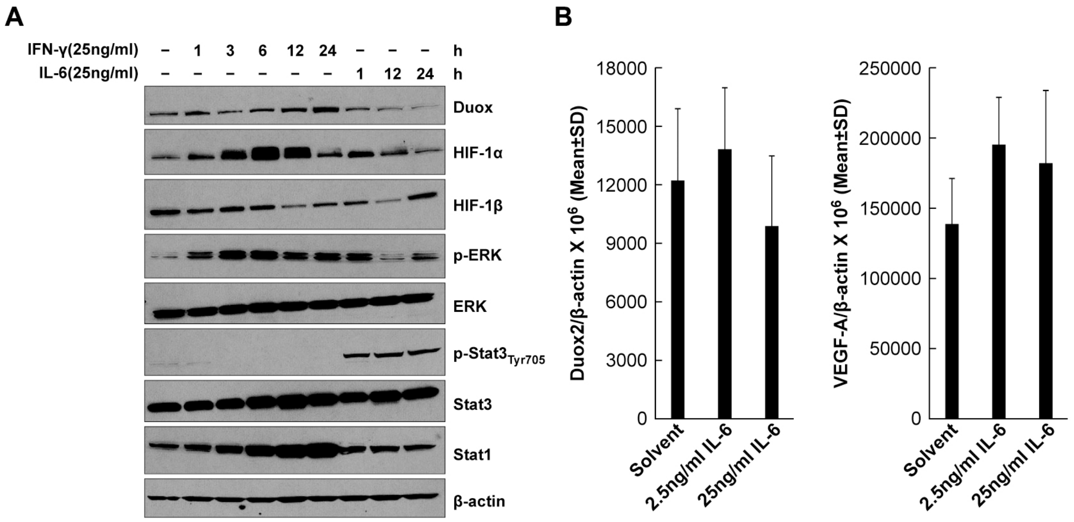

Supplementary Figure S1: Stat3 is not involved in the IFN-γ-mediated induction of VEGF-A expression in BxPC-3 cells. (**A**) Western analysis of BxPC-3 cells treated with either IFN-γ or IL-6 for the indicated times. β-actin served as the loading control. (**B**) DUOX2 (left) and VEGF-A (right) expression in BxPC-3 cells treated with IL-6 at the indicated concentrations in serum-free medium for 24 h, as determined by quantitative RT-PCR. The data are expressed as the mean ± SD of at least three independent experiments (B) or are representative of at least three independent experiments (A).

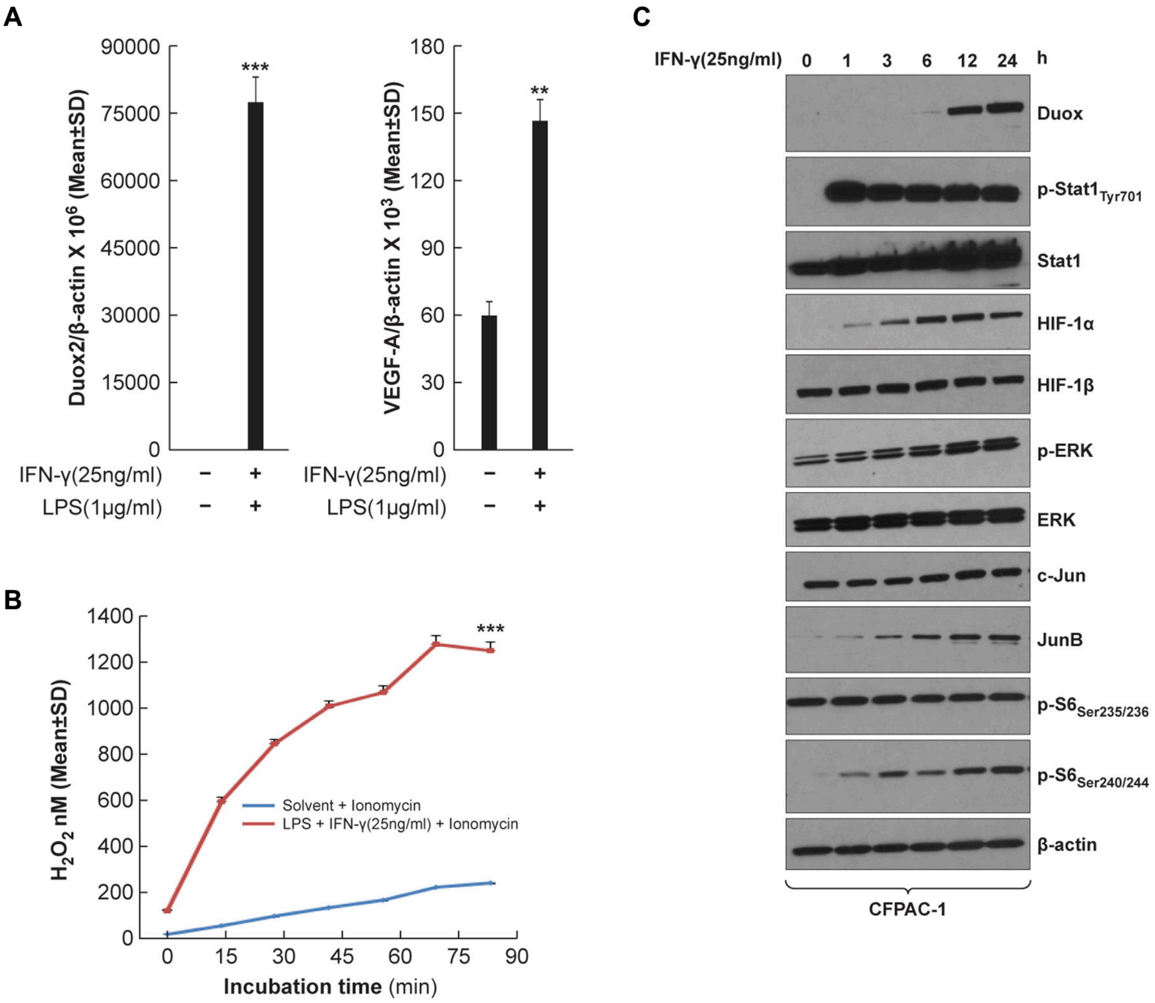

Supplementary Figure S2: CFPAC-1 pancreatic cancer cells up-regulate VEGF-A and DUOX2, produce H_2O_2, and demonstrate activation of ERK signaling and increased HIF-1α expression in response to IFN-γ and LPS. (**A**) DUOX2 (left) and VEGF-A (right) expression in CFPAC-1 cells treated with IFN-γ and LPS for 24 h, as determined by quantitative RT-PCR. $^{**}P < 0.01$, $^{***}P < 0.001$ vs. solvent-treated cells. (**B**) Extracellular H_2O_2 release in CFPAC-1 cells treated with LPS and IFN-γ or solvent for 24 h, followed by exposure to ionomycin for the indicated times. H_2O_2 values were determined based on a standard curve of 0–2 μM H_2O_2. (**C**) Western analysis of CFPAC-1 cells treated with IFN-γ for the indicated times. β-actin served as the loading control. The data are expressed as the mean ± SD of at least three independent experiments (A-B) or are representative of at least three independent experiments (C).

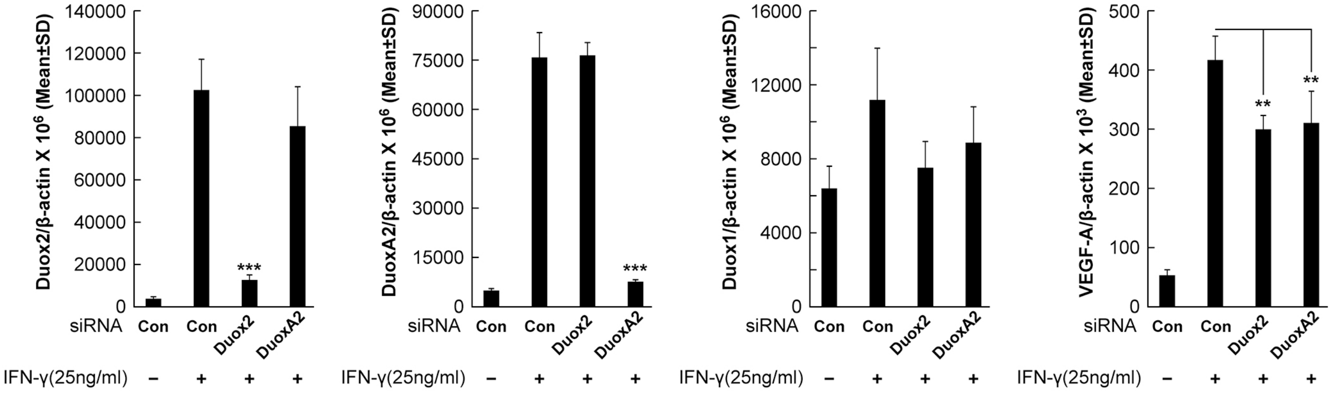

Supplementary Figure S3: Silencing of DUOX2 or DUOXA2 decreases VEGF-A expression in IFN-γ-stimulated BxPC-3 cells. Quantitative RT-PCR analysis of DUOX2, DUOXA2, DUOX1, and VEGF-A expression in BxPC-3 cells transiently transfected with control [Con], or DUOX2-, or DUOXA2-specific siRNA for 24 h, followed by IFN-γ treatment for 12 h. ***P* < 0.01, ****P* < 0.001 vs. IFN-γ-stimulated, control siRNA-treated cells. In all panels, the data are expressed as the mean ± SD of at least three independent experiments.

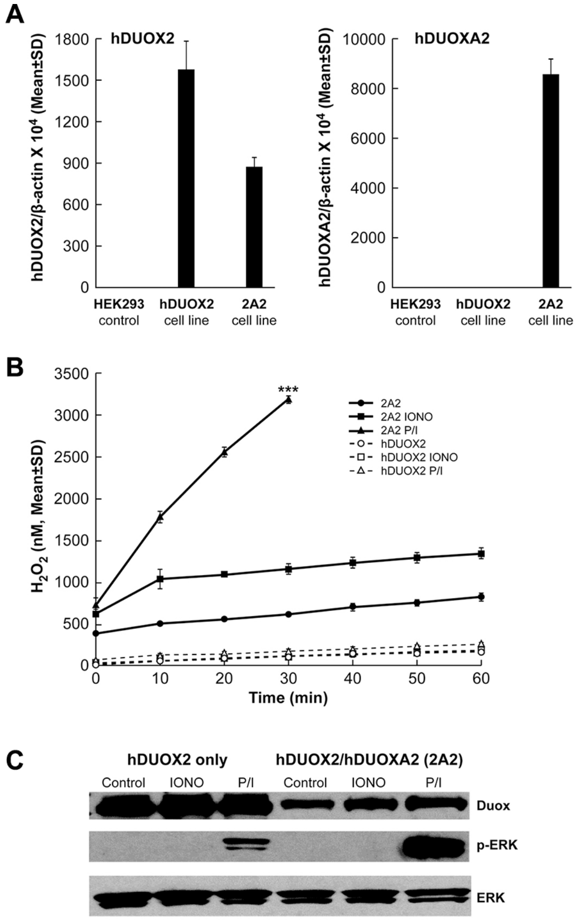

Supplementary Figure S4: Human DUOX2- and DUOXA2-expressing HEK293 cells exhibit H_2O_2 production and subsequent ERK activation in the absence of IFN-γ stimulation. (**A**) DUOX2 (left) and DUOXA2 (right) expression in HEK293 cells, human DUOX2-expressing HEK293 cells ("hDUOX2"), and human DUOX2- and DUOXA2-expressing HEK293 cells ("2A2"), as determined by quantitative PCR. (**B**) Assay of extracellular H_2O_2 production in hDUOX2 and 2A2 cells treated with or without Amplex Red® plus 1 μM ionomycin [IONO] or PMA/ionomycin [P/I] (consisting of 400 nM PMA plus 1 μM ionomycin) for the indicated times. H_2O_2 values were determined based on a standard curve of 0–2 μM H_2O_2. (**C**) Western analysis of WCEs (80 μg each) from human DUOX2-expressing or human DUOX2- and DUOXA2-expressing HEK293 cells treated with or without 100 nM ionomycin [IONO] or PMA/ionomycin [P/I] (consisting of 40 nM PMA plus 100 nM ionomycin) for 30 min. ****P* < 0.001 vs. untreated 2A2 cells, 2A2 cells exposed to ionomycin alone, or hDUOX2 cells incubated with or without P/I or ionomycin. The data are expressed as the mean ± SD of at least three independent experiments (A–B) or are representative of at least three independent experiments (C).
